# Antiviral T-Cell Frequencies in a Healthy Population: Reference Values for Evaluating Antiviral Immune Cell Profiles in Immunocompromised Patients

**DOI:** 10.1007/s10875-021-01205-1

**Published:** 2022-01-06

**Authors:** Friederike C. Schulze Lammers, Agnes Bonifacius, Sabine Tischer-Zimmermann, Lilia Goudeva, Jörg Martens, Bernd Lepenies, Maria von Karpowitz, Gunilla Einecke, Gernot Beutel, Thomas Skripuletz, Rainer Blasczyk, Rita Beier, Britta Maecker-Kolhoff, Britta Eiz-Vesper

**Affiliations:** 1 Institute of Transfusion Medicine and Transplant Engineering, Hannover Medical School, Carl-Neuberg-Str. 1, 30625 Hannover, DE Germany; 2 Institute for Immunology & Research Center for Emerging Infections and Zoonoses, University of Veterinary Medicine Hannover, Hannover, DE Germany; 3 Institute for Biostatistics, Hannover Medical School, Hannover, DE Germany; 4 Department of Nephrology, Hannover Medical School, Hannover, DE Germany; 5 Department of Hematology, Hemostasis, Oncology and Stem Cell Transplantation, Hannover Medical School, Hannover, DE Germany; 6 Department of Neurology, Hannover Medical School, Hannover, DE Germany; 7 Department of Pediatric Hematology and Oncology, Hannover Medical School, Hannover, DE Germany

**Keywords:** Antiviral T-cell repertoire, Virus-specific T cells, Adaptive immunity, Immunosuppression, Transplantation, Cellular therapy, Cellular immunity

## Abstract

Viral infections and reactivations are major causes of morbidity and mortality after hematopoietic stem cell (HSCT) and solid organ transplantation (SOT) as well as in patients with immunodeficiencies. Latent herpesviruses (e.g., cytomegalovirus, Epstein-Barr virus, and human herpesvirus 6), lytic viruses (e.g., adenovirus), and polyomaviruses (e.g., BK virus, JC virus) can cause severe complications. Antiviral drugs form the mainstay of treatment for viral infections and reactivations after transplantation, but they have side effects and cannot achieve complete viral clearance without prior reconstitution of functional antiviral T-cell immunity. The aim of this study was to establish normal ranges for virus-specific T-cell (VST) frequencies in healthy donors. Such data are needed for better interpretation of VST frequencies observed in immunocompromised patients. Therefore, we measured the frequencies of VSTs against 23 viral protein-derived peptide pools from 11 clinically relevant human viruses in blood from healthy donors (*n* = 151). Specifically, we determined the VST frequencies by interferon-gamma enzyme-linked immunospot assay and classified their distribution according to age and gender to allow for a more specific evaluation and prediction of antiviral immune responses. The reference values established here provide an invaluable tool for immune response evaluation, intensity of therapeutic drugs and treatment decision-making in immunosuppressed patients. This data should make an important contribution to improving the assessment of immune responses in immunocompromised patients.

**Supplementary Information:**

The online version contains supplementary material available at 10.1007/s10875-021-01205-1.

## Introduction

Following hematopoietic stem cell (HSCT) and solid organ transplantation (SOT), immunosuppressive therapy is administered to prevent graft rejection and graft-versus-host disease (GvHD). Prophylactic regimens transiently lead to strong immunosuppression, mainly, by decreasing CD3^+^ T-cell numbers [1]. Consequently, the risk of life-threatening bacterial, fungal, and viral infections as well as recurrent viral reactivation increases. Additionally, lymphopenia in the regeneration phase after HSCT enhances the pathogen-associated morbidity and mortality. Up to 22% and 53% of overall mortality after HSCT and SOT, respectively, are associated with infections resulting from a lack of specific T-cell immunity [2–4]. Individuals with congenital primary or secondary immunodeficiencies are even more susceptible to infectious complications, which are among the leading causes of death [5–7].

The main viral pathogens causing infection-related deaths in patients with immunodeficiency or after transplantation are endogenous herpesviruses such as cytomegalovirus (CMV), Epstein-Barr virus (EBV), and human herpesvirus 6 (HHV6); lytic viruses like adenovirus (ADV); as well as polyomaviruses such as the BK (BKV) and JC virus (JCV) [7–14]. CMV reactivation observed in 40–65% of CMV-seropositive recipients after HSCT is associated with a higher risk of mortality [15–17]. Incidence rates of EBV reactivation and post-transplant lymphoproliferative disease (PTLD) vary from 0.1 to 63%, depending on the type of transplant [17–20]. In patients with immunodeficiencies, especially severe combined immunodeficiencies, fatality rates from severe and recurrent pulmonary ADV infections as well as disseminated disease have been reported to be up to 55% [1, 21]. High-level HHV6 reactivation after allogeneic HSCT has been described in 30–50% of recipients [22, 23]. BKV and JCV viremia occur in 54% and 25% of HSCT recipients, respectively, JCV viruria in 3.8–40% of kidney transplant patients, and BKV-induced nephropathy in 5.3% of the patients [17, 24, 25]. JCV reactivation in transplant and non-transplant patients can result in life-threatening progressive multifocal leukoencephalopathy (PML), with mortality rates of up to 71% [26–28]. Other herpesviruses like herpes simplex virus type 1 (HSV1), herpes simplex virus type 2 (HSV2), and varicella-zoster virus (VZV), along with respiratory RNA viruses such as influenza A virus (IAV) and human respiratory syncytial virus (RSV), expose immunocompromised individuals to a constant risk of severe and potentially life-threatening complications [29–32].

Over the last decades, advances in antiviral drug therapy and prophylactic and pre-emptive antiviral treatment strategies have decreased infectious complications in immunocompromised patients. However, they are associated with toxic side effects and ineffective in case of drug resistances. Moreover, due to insufficient reconstitution of cellular immunity, viral infections can only be controlled but not completely eliminated [16, 33, 34]. A major clinical challenge remains the complex interplay between immunosuppressive treatment and the maintenance or establishment of antiviral immunity [8]. Therefore, clinicians must carefully balance the risks of graft rejection or GvHD on the one side and the maintenance of protective immunity on the other.

Currently, information about virus-specific T-cell (VST) frequencies required for virus control and clearance is scarce and empirical data on protective pathogen-specific T-cell numbers in blood are highly desirable. Individualized antiviral treatment strategies require knowledge about VST frequencies since it helps clinicians assess the effects of modalities such as antiviral drug therapy, and weigh the opportunity or need for reduction of immunosuppression or adoptive T-cell transfer (AT) [35–37]. If the VST frequencies of an immunosuppressed patient are within normal ranges of healthy donors, antiviral drug therapy presents a successful strategy. With VST frequencies below average, cellular therapies, such as AT, offer a promising approach. Reference ranges will help clinicians to predict responses to AT [2, 38, 39].

The aim of this study was to provide data on VST frequencies in a population of healthy donors (*n* = 151) as an aid to therapeutic decision-making in immunocompromised patients and patients with immune disorders. Antiviral T cells against 11 clinically highly relevant viruses were determined by interferon-gamma (IFN-γ) enzyme-linked immunospot (ELISpot) and characterized regarding frequency, phenotype, age, and gender. Moreover, ELISpot data were correlated to serological testing routinely performed for 7 of the 11 viruses. All donors were seropositive for at least four viruses, and the spectrum of antiviral immunity increased with age. Overall, VST frequencies were higher for DNA and persistent viruses (CMV, EBV), lower for RNA viruses (RSV, IAV), and lowest for BKV and JCV. The reference values established in this study give clinicians a valuable tool for interpreting a patient’s specific antiviral T-cell profile, and for estimating the need for and type of further therapeutic interventions, which could potentially be a breakthrough in the evaluation of immune status [40–43].

## Methods

### Study Population

VST frequencies were determined using residual blood samples from platelet apheresis disposable kits used for routine platelet collection from healthy blood donors of the Institute of Transfusion Medicine and Transplant Engineering (MHH). Written informed consent was obtained from all donors (ethics committee vote 3639–2017). Peripheral blood mononuclear cells (PBMCs) were isolated by discontinuous gradient centrifugation and resuspended in T-cell culture medium (RPMI 1640 (Lonza, Vervies) with 10% human AB serum (c.c.pro)). Whole blood and serum samples were collected on the same day for flow cytometric and serological analysis.

### Flow Cytometry

Whole blood samples were transferred into BD Trucount™ tubes and extracellularly stained (Tab.S1), followed by erythrocyte lysis (Lysing Solution, BD Biosciences). For analysis of IFN-γ ELISpot assay﻿, 1 × 10^6^ isolated PBMCs were extracellularly stained (Tab.S2) and washed. Samples were acquired on a FACSCanto™ 10c cytometer (BD Biosciences), and data were analyzed with BD FACSDiva™ Software v8.0. Gating strategies for CD3^+^, CD4^+^, and CD8^+^ T cells as well as for memory T-cell phenotypes are shown in Fig.S1A + B.

### IFN-γ ELISpot

IFN-γ ELISpot assay was performed as described previously [44, 45]; details are provided in supplementary information (Methods, Tab.S3).

### Serology

CMV-specific antibodies were detected using CMV-IgG assay (Abbott Diagnostic). Additionally, antibodies against CMV, EBV, and HSV1/2 (recomLine, Mikrogen) as well as ADV, VZV, RSV, and IAV (NovaLisa, NovaTec Immundiagnostica GmbH) were analyzed.

### Data Analysis

Data were analyzed using Microsoft Excel 2010/2016 (Microsoft Corporation) and displayed using FlowJo™ v10 (FlowJo™ LLC, BD Biosciences) and GraphPad Prism v8.2 (GraphPad Software).

## Results

### Donor Cohort

A total of 151 healthy donors (87 male, 64 female) with an average age of 42 years (19–67 years) were included in this study (Fig.S1C). The population was divided into four groups according to age and gender: males 40 years of age or younger (*n* = 40), males over the age of 40 (*n* = 47), females 40 years of age or younger (*n* = 30), and females over the age of 40 (*n* = 34). Frequencies of naïve T cells (T_N_: CD45RA^+^CD62L^+^) were higher in younger donors, while central memory T cells (T_CM_: CD45RA^−^CD62L^+^) were more frequent among older donors (Fig.S1D). Slightly higher frequencies of effector memory T cells (T_EM_: CD45RA^−^CD62L^−^) were observed in males than in females. Frequencies of CD4^+^ T effector memory cells re-expressing CD45RA (T_EMRA_: CD45RA^+^CD62L^−^) were lower than CD8^+^ T_EMRA_ in all groups.

Routinely applied serological testing was performed to determine the serostatus for CMV, EBV, HSV, VZV, ADV, RSV, and IAV (Tab.S4). Initially, 76 donors (50.3%) were classified as CMV-seropositive by routine anti-CMV-IgG ELISA testing, which was not confirmed in 10 of these donors by immunoblot [44]. Since follow-up samples confirmed the negative serology, further analysis was based on immunoblot results. Interestingly, within CMV- and HSV-seropositive subgroups, the proportion of EBV-seropositive donors (95.5% and 96.9%) was increased compared with the overall cohort. Similarly, the proportion of HSV-seropositive donors within CMV-seropositive donors (75.8%) was increased compared with the overall cohort. All donors were seropositive for at least four of the seven tested viruses and the number of positive serological tests increased with age (Tab.S5).

### Antiviral T-Cell Frequencies

**Fig. 1:**
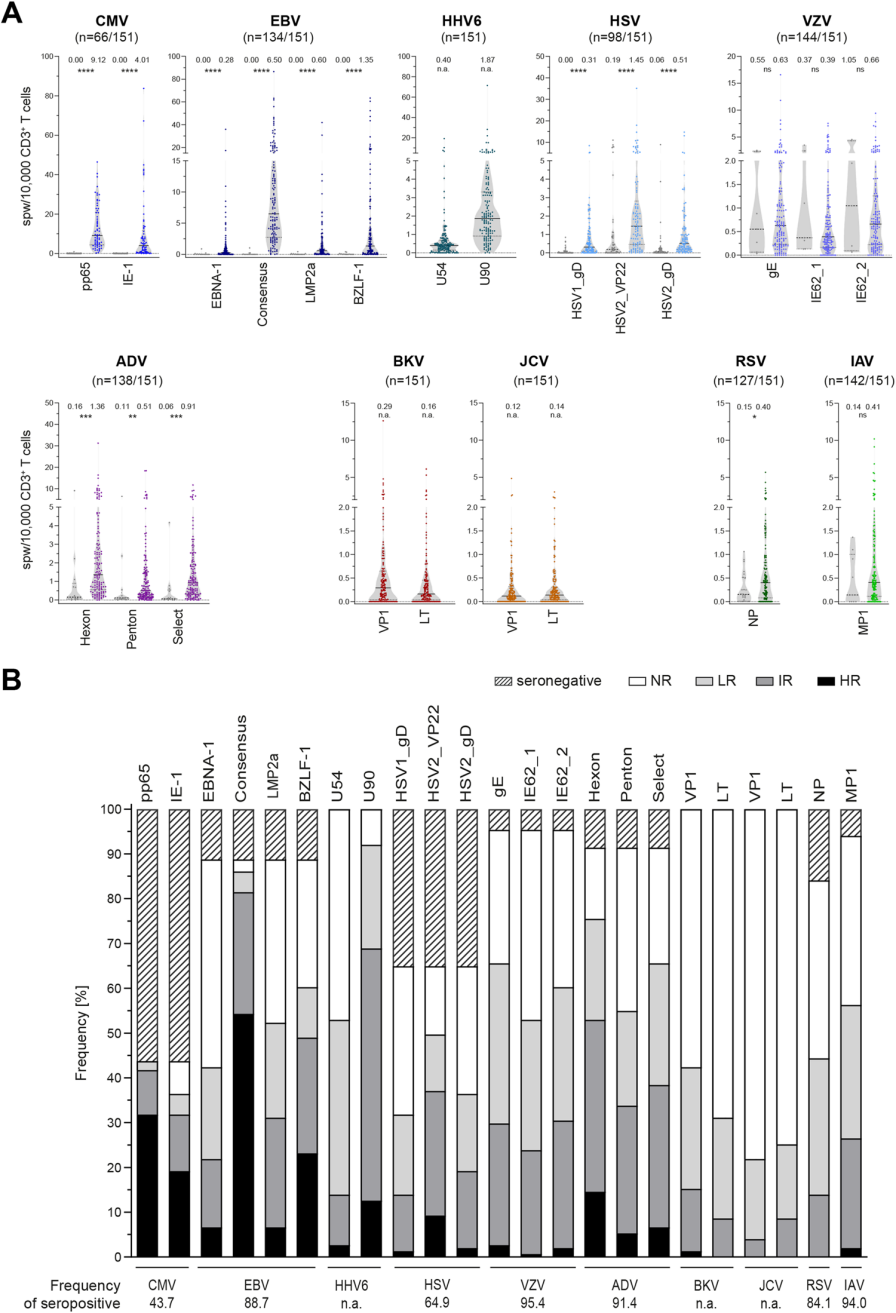
Antiviral T-cell frequencies in seropositive donors. Frequencies of antiviral T-cells were determined in a large cohort of healthy donors (*n* = 151) by ELISpot assay. **A** Antiviral T-cell frequencies normalized to CD3^+^ T-cell frequencies within PBMCs and expressed as spots per 10,000 CD3^+^ T cells in seropositive and seronegative donors (colored and gray symbols, respectively), unless otherwise stated. Data are shown as violin plots; each symbol represents one donor. Horizontal lines represent median values, and dotted lines the 25% and 75% percentiles, respectively. The number above each data set indicates the median. Asterisks show significant difference to values obtained from seronegative donors (Mann–Whitney test). **B** Comparative overview of frequency of donors with positive serology for which virus-specific T cells for the respective antigens were detected (total number of donors: *n* = 151). Donors were grouped according to the number of spots per well (spw) generated in response to each peptide pool as follows: high responders (HR, ≥ 50spw or 47spw + 2 × negative control (NC)), intermediate responders (IR, ≥ 10spw or 7spw + 2xNC), low responders (LR, ≥ 3spw or 2xNC), and non-responders (NR, < 3spw or 2xNC). CMV cytomegalovirus, EBV Epstein-Barr virus, HHV6 human herpesvirus 6, HSV herpes simplex virus, VZV varicella-zoster virus, ADV adenovirus, BKV BK polyomavirus, JCV JC polyomavirus, RSV respiratory syncytial virus, IAV influenza A virus. * *p* < 0.05, ** *p* < 0.01, *** *p* < 0.001, **** *p* < 0.0001, ns not significant, n.a. not applicable

**Fig. 2:**
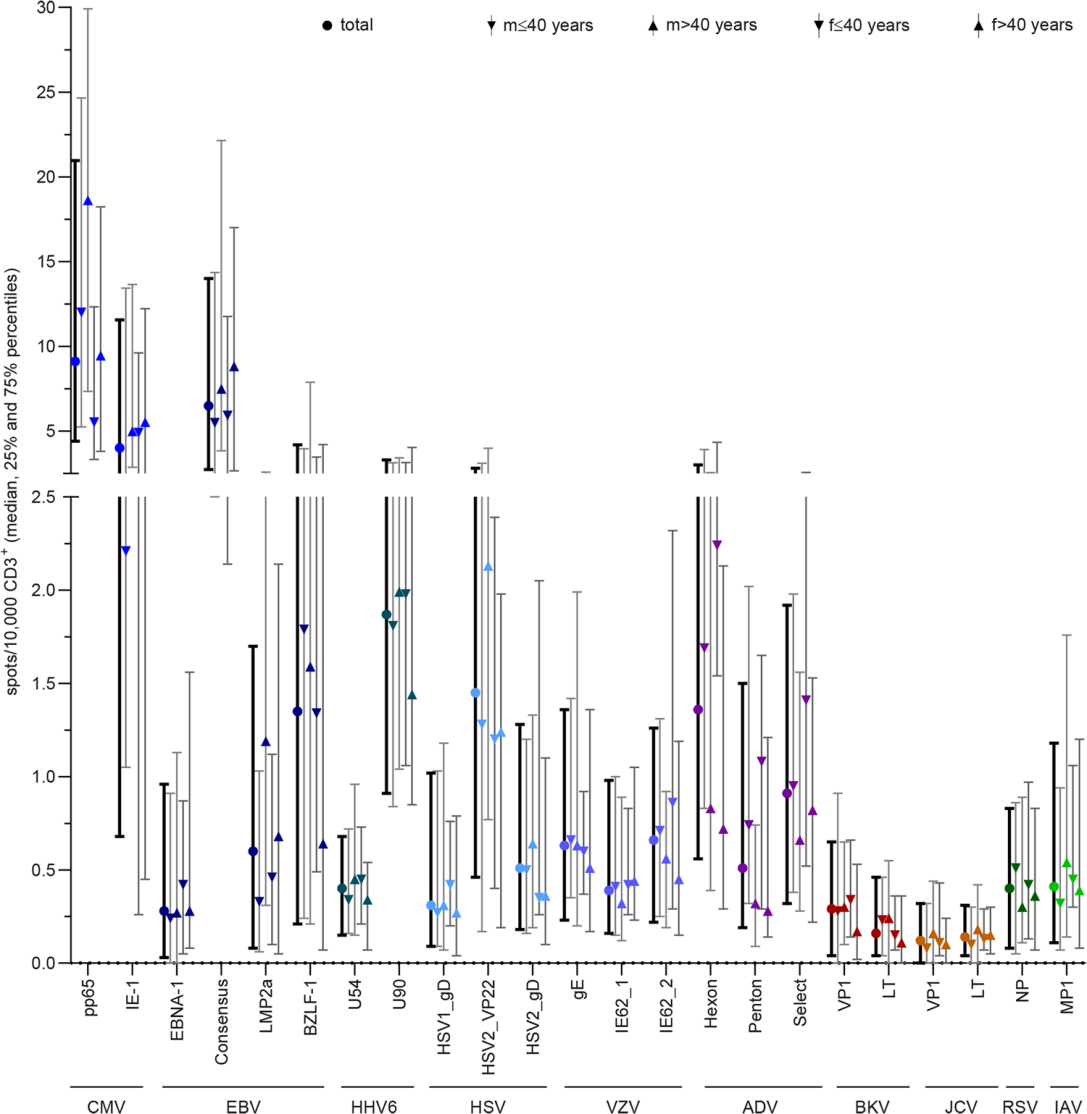
Reference values for males (m) aged ≤ 40 and > 40, and females (f) aged ≤ 40 and f > 40. Antiviral T-cell frequencies in *n* = 151 healthy donors, normalized to CD3^+^ T-cell frequencies within PBMCs and expressed as spots per 10,000 CD3^+^ T cells, as measured in all (HHV6, BKV, JCV) or seropositive donors (CMV, EBV, HSV, VZV, ADV, RSV, IAV) (see also Fig. 1). Symbols represent median values, and vertical lines the 25% and 95% percentiles. See also Tab. S3. CMV cytomegalovirus, EBV Epstein-Barr virus, HHV6 human herpesvirus 6, HSV herpes simplex virus, VZV varicella-zoster virus, ADV adenovirus, BKV BK polyomavirus, JCV JC polyomavirus, RSV respiratory syncytial virus, IAV influenza A virus

VST frequencies against 23 peptide pools derived from 11 viruses were determined by IFN-γ ELISpot and normalized to CD3^+^ T-cell numbers and used to classify donors as non-, low, intermediate, and high responders (Fig. 1). Antiviral T-cell frequencies were analyzed regarding age and gender distribution, CD3^+^ T-cell frequencies, and the number of functionally active VSTs per microliter blood (Figs.S1C+D, S2-S11). Reference values for evaluation of immune responses in patients were generated by calculating mean ± SD, minimum, maximum, 25% and 75% percentiles, median, and limits of the 95% confidence interval (Tables 1 and S6, Figs. 1A and 2).

**Table 1:** Reference values based on serological results and frequencies of virus-specific T-cells in healthy donors (*n* = 151). See also Tab. S3

Virus	Antigen	Min	25% percentile	Median	75% percentile	Max	Mean	SD	Lower 95% CI of median	Upper 95% CI of median
Seropositive out of total *n* (%)										
**CMV** 66 (43.7)	pp65	0.25	4.41	9.12	20.97	46.42	13.17	11.18	10.43	15.92
	IE-1	0.00	0.68	4.01	11.56	83.76	10.32	16.46	6.28	14.37
**EBV** 134 (88.7)	EBNA-1	0.00	0.03	0.28	0.96	35.90	1.31	3.75	0.67	1.95
	Consensus	0.00	2.73	6.50	14.01	86.61	11.69	14.22	9.26	14.12
	LMP2a	0.00	0.08	0.60	1.70	41.83	1.83	4.80	1.01	2.65
	BZLF-1	0.00	0.21	1.35	4.19	63.34	5.38	11.04	3.50	7.27
**HHV6**	U54	0.00	0.15	0.40	0.68	19.25	0.82	1.99	0.50	1.14
	U90	0.00	0.91	1.87	3.31	71.34	3.20	6.61	2.14	4.27
**HSV** 98 (64.9)	HSV1_gD	0.00	0.09	0.31	1.02	8.47	0.80	1.26	0.55	1.05
	HSV2_VP22	0.00	0.46	1.45	2.81	35.12	2.82	4.60	1.89	3.74
	HSV2_gD	0.00	0.18	0.51	1.28	14.78	1.16	2.15	0.73	1.59
**VZV** 144 (95.4)	gE	0.00	0.23	0.63	1.36	16.58	1.20	2.02	0.86	1.53
	IE62_1	0.00	0.16	0.39	0.98	7.54	0.90	1.34	0.68	1.12
	IE62_2	0.00	0.22	0.66	1.26	9.43	1.08	1.48	0.83	1.32
**ADV** 138 (91.4)	Hexon	0.00	0.56	1.36	3.01	31.23	2.67	3.86	2.02	3.32
	Penton	0.00	0.19	0.51	1.50	18.40	1.40	2.62	0.96	1.84
	Select	0.00	0.32	0.91	1.92	11.77	1.52	1.94	1.19	1.84
**BKV**	VP1	0.00	0.04	0.29	0.65	12.62	0.64	1.30	0.43	0.85
	LT	0.00	0.04	0.16	0.46	6.14	0.44	0.85	0.31	0.58
**JCV**	VP1	0.00	0.00	0.12	0.32	4.86	0.29	0.55	0.20	0.38
	LT	0.00	0.04	0.14	0.31	3.04	0.32	0.51	0.24	0.40
**RSV** 127 (84.1)	NP	0.00	0.08	0.40	0.83	5.69	0.67	0.91	0.51	0.83
**IAV** 142 (94.0)	MP1	0.00	0.11	0.41	1.18	10.20	0.96	1.58	0.70	1.22

*CMV* cytomegalovirus, *EBV* Epstein-Barr virus, *HHV6* human herpesvirus 6, *HSV* herpes simplex virus, *VZV* varicella-zoster virus, *ADV* adenovirus, *BKV* BK polyomavirus, *JCV* JC polyomavirus, *RSV* respiratory syncytial virus, *IAV* influenza A virus

#### Herpesviruses

All *CMV*-seropositive donors had T-cell responses against phosphoprotein 65 (pp65). CMV_pp65-specific T-cell frequencies were higher than those for immediate early protein-1 (IE-1) (Figs. 1, 2, S2, Tables 1 and S6). In CMV-seronegative donors, no CMV-specific T cells were detected. Frequencies of CMV-specific T cells slightly increased with age in males, but not in females. The frequency of memory T cells was markedly higher in CMV-seropositive than in CMV-seronegative donors and mainly consisted of T_EMRA_ (Tab.S7). Interestingly, the percentage of total CD3^+^ T_EMRA_ within the different responder groups was larger in high responders than in intermediate and low responders.

All *EBV*-seropositive donors had detectable EBV-specific T cells against at least one of the peptide pools Epstein-Barr nuclear antigen-1 (EBNA-1), Consensus, latent membrane protein 2a (LMP2a), and BamHI Z fragment leftward open reading frame 1 (BZLF-1) (Figs. 1, 2, S3, Tables 1 and S6). The remaining EBV-seronegative donors had no detectable EBV-specific T cells. Moreover, 97.0% of the EBV-seropositive donors possessed EBV_Consensus-specific T cells, while less donors responded to the other tested antigens. Most high responders were found for EBV_Consensus (63.1%) and EBV_BZLF-1 (38.5%). Likewise, antigen-specific T cells to EBV_Consensus were most frequent, and their frequency slightly increased with age regardless of gender. Moreover, LMP2a-specific T-cell frequencies increased significantly with age in males. EBV_Consensus low and non-responders had noticeably increased frequencies of CD8^+^ T_N_ cells accompanied by lower frequencies of CD8^+^ T_CM_ compared to high and intermediate responders (Tab.S7). Accordingly, EBV_LMP2a intermediate, low, and non-responders had higher frequencies of CD8^+^ T_N_ than high responders. T_N_ frequencies generally decreased from non- to high responders, while T_CM_ frequencies increased.

*HHV6*_U90-specific T cells were detected in 139/151 donors (92.1%), most (61.2%) of whom were intermediate responders, while HHV6_U54-specific T cells were detected in only 80/151 donors (53.0%), who were mostly low responders (*n* = 59/80, 73.8%) (Figs. 1, 2, S4, Tables 1 and S6). In line with that, the frequency of HHV6_U54-specific T cells was markedly lower than that of HHV6_U90-specific T cells. Younger and older men had comparable HHV6-specific T-cell frequencies, while HHV6_U90-specific T-cell frequencies marginally decreased with age in women. Both intermediate and high responders had lower frequencies of T_N_ and higher frequencies of T_EMRA_ than low and non-responders (Tab.S7).

Among the 53 *HSV*-seronegative donors (35.1%), 21 donors (39.6%) had HSV-specific T cells. Moreover, among the 98 donors (64.9%) classified as HSV-seropositive, no HSV-specific T cells could be detected in nine donors (9.18%). Only HSV-seropositive donors were included in T-cell response analysis (Figs. 1, 2, S5, Tables 1 and S6). The highest response rate was observed for HSV2 tegument protein VP22 (VP22; 76.5%), followed by HSV2 envelope glycoprotein D (gD) (56.1%) and HSV1_gD (49.0%). In line with that, high responders were the most frequent kind of HSV2_VP22 responders (18.7%). Highest frequencies of HSV-specific T cells were observed for HSV2_VP22, followed by HSV2_gD and HSV1_gD. In male donors, HSV2_VP22-specific T-cell frequencies slightly increased with age. High responders had markedly higher frequencies of T_EMRA_ than the other responder groups (Tab.S7).

A large fraction of donors was *VZV*-seropositive (*n* = 144/151, 95.4%; Fig. 1B). In three donors identified as VZV-seronegative, VZV-specific T cells were detected (*n* = 3/7; 42.9%). Only VZV-seropositive donors were included in T-cell response analysis (Figs. 1, 2, S6, Tables 1 and S6). A large proportion of VZV-seropositive donors did not have detectable VZV-specific T cells (87.5%). VZV envelope glycoprotein E (gE) elicited the highest responder rate (68.8%). Most donors were classified as intermediate or low responders. Overall, responder group distributions were comparable for all three peptide pools, as were frequencies of VZV-specific T cells. No distinct differences were observed in relation to age and gender distribution. Most intermediate responders had lower frequencies of T_N_ than low and non-responders (Tab.S7).

#### Adenovirus

A total of 138 donors (91.4%) were tested *ADV*-seropositive by ELISA covering ADV type 2. Fifteen of these individuals (10.9%) showed no response to any of the ADV peptide pools (ADV5_Hexon, ADV5_Penton, ADV2/5_Select), while ADV-specific T cells were detected in four ADV-seronegative donors (30.8%). These were mainly reactive against ADV5_Hexon and ADV5_Penton. Only seropositive donors were included in T-cell response analysis (Figs. 1, 2, S7, Tables 1 and S6). Most ADV-specific T cells were found to be specific for ADV5_Hexon (82.6%), followed by ADV2/5_Select (71.7%) and ADV5_Penton (60.1%). Likewise, most high responders were found for ADV5_Hexon (19.3%), and the highest ADV-specific T-cell frequencies were found for ADV5_Hexon. ADV_Penton- and ADV_Hexon-specific T-cell frequencies decreased with age in males and females, respectively. The four responder groups had comparable T-cell phenotype distributions (Tab.S7). However, ADV5_Penton high responders had less CD8^+^ T_N_ and more T_EM_ than intermediate, low, and non-responders.

#### Polyomaviruses

Sixty-four (42.4%) donors responded to *BKV*_major capsid protein (VP1) and 47 (31.1%) responded to BKV large T antigen (LT), including only 2/64 (3.13%) high responders to BKV_VP1 and no high responders to BKV_LT (Figs. 1, 2, S8, Tables 1 and S6). BKV_VP1-specific T-cell frequencies were higher than BKV_LT-specific T-cell frequencies. Females had slightly lower frequencies of BKV_LT-specific T cells than males, and their overall BKV-specific T-cell frequencies tend to decrease with age. Low and non-responders had lower numbers of T_EMRA_ and higher frequencies of T_CM_ than intermediate responders (Tab.S7).

None of the 33 *JCV*_VP1 (21.9%) or 38 JCV_LT responders (25.2%) were high responders (Figs. 1, 2, S9, Tables 1 and S6). Furthermore, JCV had the lowest number of responders and lowest VST frequencies of all tested viruses. JCV-specific T-cell frequencies appear to be highest in older males. Interestingly, JCV_VP1 intermediate responders had markedly increased frequencies of T_EMRA_ cells compared to non- and low responders, while JCV_LT intermediate responders had only slightly higher percentages of CD4^+^ T_EMRA_ than non- and low responders (Tab.S7).

#### RNA Viruses

By ELISA, 127/151 donors (84.1%) were classified as *RSV*-seropositive; RSV-specific T cells were detected in 8 (33.3%) RSV-seronegative donors. Only RSV-seropositive donors were included in T-cell analysis (Figs. 1, 2, S10, Tables 1 and S6). Most RSV-seropositive donors had little or no detectable specific T-cell response against RSV nucleoprotein (NP): 47.2% were non- and 36.2% low responders. RSV-specific T-cell frequencies marginally decreased with age. Interestingly, intermediate responders had slightly higher fractions of *T*_CM_ and *T*_EMRA_ than low and non-responders (Tab.S7).

One hundred forty-two of 151 donors (94.0%) were identified as *IAV*-seropositive by ELISA. IAV-specific T cells against IAV matrix protein 1 (MP1) were detected in 2/9 (22.2%) IAV-seronegative donors. Only IAV-seropositive donors were included in T-cell evaluation (Figs. 1, 2, S11, Tables 1 and S6). T cells were detected in 59.9% of seropositive donors, who were mainly low (31.7%) and intermediate responders (26.1%). IAV-specific T-cell frequencies slightly increased with age in males. High responders (2.11%) had markedly lower frequencies of *T*_N_ and higher frequencies of *T*_CM_ (Tab.S7).

### Summary and Correlations of Antiviral T Cells

**Fig. 3:**
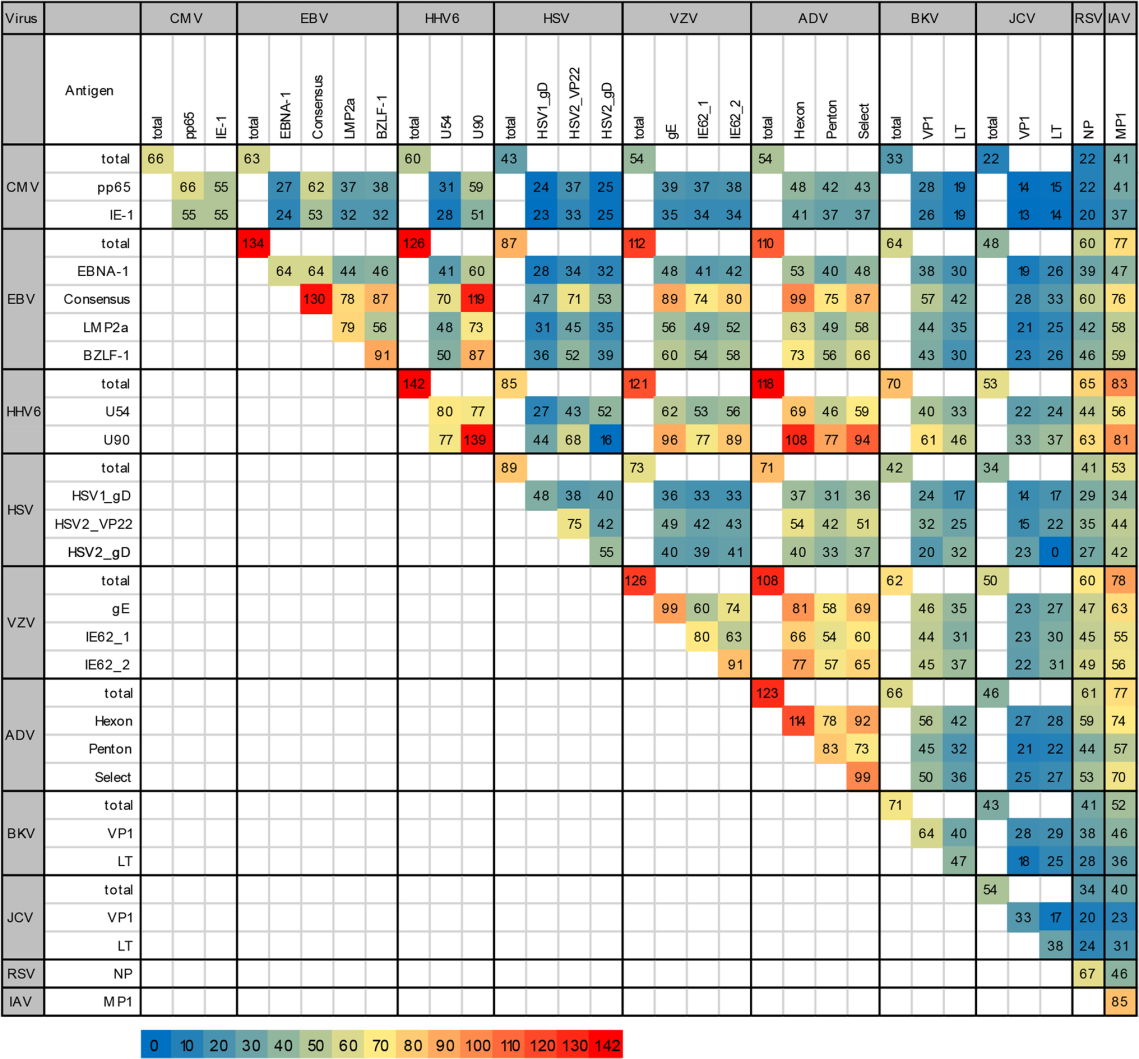
Relation between virus-specific T cells (VSTs). Number of donors with detectable VSTs against the indicated combinations of viral antigens. Where applicable, only seropositive donors were included (see also Fig. 1B). CMV cytomegalovirus, EBV Epstein-Barr virus, HHV6 human herpesvirus 6, HSV herpes simplex virus, VZV varicella-zoster virus, ADV adenovirus, BKV BK polyomavirus, JCV JC polyomavirus, RSV respiratory syncytial virus, IAV influenza A virus

In the total cohort, a small fraction of donors had T cells against CMV, BKV, and JCV while more donors responded to EBV, ADV, HHV6, and VZV (Fig. 1B). All CMV-seropositive donors had T-cell responses to CMV_pp65. For the remaining viruses tested, no VSTs were detectable in a large fraction of seropositive donors except EBV (Consensus; 97.0%). Furthermore, most donors had T cells against HHV6 (U90; 92.1%). CMV- and EBV-specific T-cell frequencies appeared to increase with age, while ADV-specific T-cell frequencies decreased, regardless of gender (Fig. 2). Figure 3 shows the numbers of donors with antiviral T cells against each viral antigen in parallel. Interestingly, few donors had VSTs against both BKV peptide pools (*n* = 40/71 responders; 56.3%). This was even more pronounced for JCV (*n* = 17/54 responders; 31.5%). Despite high sequence homology, considerable differences in responder rates presented for BKV and JCV. However, 28/64 (43.8%) BKV_VP1 responders also responded to JCV_VP1, and 25/47 (53.2%) BKV_LT responders also responded to JCV_LT. While for some viruses, serological testing was not in line with VST frequencies, the previously described serological correlations between CMV, EBV, and HSV were partly reflected at the T-cell level.

## Discussion

Immunocompromised HSCT and SOT recipients and individuals with congenital primary or secondary immunodeficiencies present a high burden of mortality due to life-threatening bacterial, fungal, and viral infections as well as recurrent viral reactivations [1, 5, 7]. Although antiviral drug treatments have advanced over the years, they are still associated with toxic side effects. In the absence of functional VSTs, they can only control but not completely eliminate viruses [16, 33, 34]. Antiviral T-cell frequencies determine whether treatment (e.g., antiviral drug therapy, reduction of immunosuppression, or AT) is needed [35–37]. This study aimed to establish reference values for antiviral T-cell frequencies against 11 clinically relevant human viruses in healthy donors. These data should help to improve the prevention and treatment of viral complications, leading to better outcomes in HSCT and SOT recipients and patients with immune disorders.

In the transplant setting, determination of infectious disease markers for viruses mainly includes the CMV and EBV serostatus of donor and recipient. Donor seropositivity provides an opportunity for transfer of antigen-specific T cells to improve immunity in HSCT recipients with insufficient endogenous antiviral immunity. Conversely, SOT from seropositive donors to negative recipients is associated with an increased risk of primary viral infections in immunosuppressed SOT recipients. In this study, the discrepancies between ELISA-based CMV-IgG assay and immunoblotting as well as ELISpot assay for CMV indicated a false-positive rate of 13.1% for the CMV-IgG assay. As confirmed by other studies, standard serology tests might not be reliable enough for adoptive immunotherapy when no peptide-specific memory T cells are present [44, 46]. Contrary to other studies [44, 47], we did not detect any CMV-specific T cells in seronegative donors, but the lack of detection might be due to the relatively short restimulation. Moreover, some donors with VSTs against a given virus were categorized as seronegative by ELISA, particularly for ADV, HSV, VZV, RSV, and IAV, which might be due to utilization of different, less concentrated surface antigens in ELISA compared to ELISpot. For instance, here, the utilized ADV-IgG assay covers ADV type 2 while ADV5_Hexon and ADV5_Penton peptide pools are derived from ADV type 5. Conversely, it might explain the lack of functional VSTs in some donors identified as ADV-seropositive. Similar circumstances presented for HSV, VZV, RSV, and IAV. Overall, majority of seropositive donors had the respective VSTs and their frequencies were significantly higher compared to seronegative donors. Furthermore, T-cell and antibody production is dependent on reinfection and recall immunization, which might not have occurred in these donors. However, one study using the HSV immunoblot assay suggests that exposure to HSV can induce HSV-specific cellular immunity without seroconversion [48]. This may also explain the observed discrepancy between HSV serostatus and the detection of HSV-specific T cells.

High frequencies of herpesvirus-specific T cells were detected in the present study, suggesting that viruses causing persistent infections are able to generate higher T-cell frequencies. Others have observed comparably high frequencies of VSTs for CMV [49] and EBV in healthy seropositive donors [50]. The ability of these viruses to achieve latency and frequently initiate productive replication cycles was shown in murine models [51]. This mechanism provides a continuous stimulus for the maintenance of VSTs. The positive correlation within the group of herpesviruses found in this study has been demonstrated previously [52–55]. It has been hypothesized that their high co-prevalence is associated with increased age or lower socioeconomic status [52]. Our study confirmed the association between age and seroprevalence, but did not assess socioeconomic factors. The majority of EBV-seropositive donors in the present study had EBV_Consensus-specific T cells. In contrast to the EBV-derived overlapping peptide pools covering the entire sequence of the respective protein, Consensus contains a mix of peptides derived from 13 lytic and latent EBV proteins, and covers 14 frequent HLA class I and II molecules. The use of overlapping peptide pools allows for detection of T-cell responses to multiple epitopes regardless of HLA type [56, 57], while the use of an antigen pool derived from various proteins with different HLA restrictions leads to a greater T-cell response with a higher range of clinically relevant VSTs due to its high antigenic diversity [58]. EBNA1-specific T cells are of utmost clinical importance since EBNA-1 plays numerous roles in EBV latency and is the only EBV protein expressed in all EBV-associated tumors [59, 60]. JC polyomavirus, which establishes persistent infections in the kidney and lymphoid organs, normally remains dormant but can reactivate in immunocompromised individuals where it can cause PML, a life-threatening infection of the brain [26–28, 61]. In this study, JCV yielded the lowest VST frequencies of all viruses tested. JCV-specific VST frequencies were reported to be low, but without sufficient evidence [62, 63]. For IAV, a virus not typically causing long-term latent or persistent infections, we observed higher frequencies of antigen-specific T cells than for BKV and JCV. This finding contradicts the notion of a correlation between general viral latency and higher immune response. Herpesviruses are among the few viruses capable of true latency, i.e., persistence and reversibility, characterized by reactivation of expression of the entire viral genome under certain conditions [64]. The results of the present study suggest that true viral latency is associated with the generation of higher VST frequencies.

In this study, IAV and RSV were characterized by low VST frequencies. Both are RNA viruses infecting cells by directly releasing RNA into the cytoplasm of host cells [65]. Once inside, viral proteins can be replicated without transcribing viral DNA into RNA, unlike DNA viruses. Many RNA viruses do not elicit long-lasting immune protection after infection due to their innate immune evasion strategies and can cause a reoccurrence of symptoms. While IAV is able to elicit protective immunity, its genetic drift and shift usually lead to inadequate immune responses after reinfection. Because the affected immune response also impacts subsequent adaptive responses, viral innate immune evasion often undermines fully protective immunity [66].

Even though BKV belongs to the group of viruses with generally low VST frequencies [67], it is a major complication after kidney transplantation and therefore of high clinical relevance [17, 24, 68]. Despite high sequence homology between BKV and JCV, VST frequencies for JCV were lower compared to BKV. In line with previous studies, we observed a correlation between BKV- and JCV-reactive T cells [69, 70], implicating high potential of BKV-specific T cells for treatment of both BKV- and JCV-associated diseases such as PML, where third-party BKV-specific T-cell transfer has shown promising results [28, 71, 72].

The phenotypic structure of VSTs involved in different viral infections and reactivations varies due to differences in response patterns. In our study, T_N_ frequencies were generally higher in individuals lacking antiviral T cells and in seronegative donors. It has been reported that while EBV- and HSV-specific T cells included higher ratios of CD8^+^ T cells, CD4^+^ T cells were the dominant T-cell subset in ADV, BKV, and VZV [73–77]. The importance of T-cell subsets varies depending on the virus and the associated disease and needs to be considered when evaluating patient immune status.

Our findings confirmed that antiviral immunity increases with age and that seroprevalence is higher among older individuals [78]. While age is associated with a highly differentiated T-cell repertoire because the cumulative number of contacts to viral agents increases over time [79], the process of immunosenescence leads to an age-related decrease in immune system activity, including T-cell function, despite higher effector T-cell frequencies [78, 80, 81]. Consequently, the decline in immune function is believed to increase the risk of viral infections and reactivations, leading to higher mortality rates among the elderly [40, 82]. Here, we determined the frequencies of IFN-γ-producing, functional, antiviral T cells, thereby—at least in part—accounting for the possible loss of immune function associated with aging. However, our cohort did not cover the entire age range. Characterization of the antiviral T-cell repertoire of older individuals requires the inclusion of additional factors like T-cell senescence, exhaustion, and additional effector molecules. However, as corroborated by other studies, our results indicate a correlation between age and VST frequency, possibly caused by more frequent and/or recurrent viral infections over time [53, 83, 84]. In contrast, younger individuals with no history of exposure to a broad variety of antigens often suffer from severe viral infections after transplantation due to the lack of endogenous antiviral T cells. In particular, EBV causes PTLD in many young patients after transplantation [20, 85]. Due to their limited antiviral T-cell repertoire, further studies are needed to determine the VST frequencies for adjusting antiviral or immunosuppressive treatment strategies in young patients.

This study aimed to improve the clinical applicability of antiviral T-cell frequencies by characterizing T cells specific to clinically relevant viruses in terms of numbers as well as age and gender distribution in a great cohort of healthy donors. In line with previous studies, this study demonstrated that antiviral immunity increases with age. Furthermore, a positive correlation within herpesviruses was found. With exception of CMV_pp65, positive serology was not necessarily equivalent to detection of the respective VSTs. The findings of this study have important implications for the evaluation of T-cell mediated immunity and treatment decision-making to determine the need for antiviral treatment or reduction of immunosuppression. Together, this data will improve the outcome of immunocompromised patients and provide better comparability of currently used immunogenic stimulants regarding clinical outcome.

## Supplementary Information

Below is the link to the electronic supplementary material.

Supplementary file1 (DOCX 2.11 MB)

## Data Availability

All data generated in this study are provided in the main manuscript and supplemental information.
