# Supplementary material for: Antiviral T-Cell Frequencies in a Healthy Population: Reference Values for Evaluating Antiviral Immune Cell Profiles in Immunocompromised Patients
Source: J Clin Immunol. 2022 Jan 6;42(3):546–58. doi: 10.1007/s10875-021-01205-1 (PMC9015970; doi:10.1007/s10875-021-01205-1)
Supplement: Supplementary file 1 — Supplementary file1 (DOCX 2.11 MB) [file 10875_2021_1205_MOESM1_ESM.docx]

**Antiviral T-Cell Frequencies in a Healthy Population: Reference Values for Evaluating Antiviral Immune Cell Profiles in Immunocompromised Patients**

Friederike C. Schulze Lammers^1^, Agnes Bonifacius^1^, Sabine Tischer-Zimmermann^1^, Lilia Goudeva^1^, Jörg Martens^1^, Bernd Lepenies^2^, Maria von Karpowitz^3^, Gunilla Einecke^4^, Gernot Beutel^5^, Thomas Skripuletz^6^, Rainer Blasczyk^1^, Rita Beier^7^, Britta Maecker-Kolhoff^7^, Britta Eiz-Vesper^1*^

^1^ Institute of Transfusion Medicine and Transplant Engineering, Hannover Medical School, Carl-Neuberg-Str. 1, 30625, Hannover, DE, Germany

^2^ Institute for Immunology & Research Center for Emerging Infections and Zoonoses, University of Veterinary Medicine Hannover, Hannover, DE, Germany

^3^ Institute for Biostatistics, Hannover Medical School, Hannover, DE, Germany

^4^ Department of Nephrology, Hannover Medical School, Hannover, DE, Germany

^5^ Department of Hematology, Hemostasis, Oncology and Stem Cell Transplantation, Hannover Medical School, Hannover, DE, Germany

^6^ Department of Neurology, Hannover Medical School, Hannover, DE, Germany

^7^ Department of Pediatric Hematology and Oncology, Hannover Medical School, Hannover, DE, Germany

Friederike C. Schulze Lammers and Agnes Bonifacius contributed equally to this work.

*Corresponding author email: eiz-vesper.britta@mh-hannover.de

**Supplementary Methods**

**IFN-γ ELISpot**

IFN-γ ELISpot assay was performed as follows: 2.5×10^5^ PBMCs/well (after overnight resting in TCM) were seeded in a 96-well plate and stimulated with 1 µg/ml staphylococcus enterotoxin B (SEB) (positive control, Sigma-Aldrich, Hamburg), 1 µg per peptide/ml for peptide pools of the respective antigens (Table S3) or without antigen (negative control, NC). Following overnight incubation, IFN-γ secretion was detected and data were acquired on an ‘AID iSpot Reader System’ (AID GmbH, Straßberg). Spot counting was performed with ‘AID ELISpot Software Version 8.0′ (https://www.aid-diagnostika.com). All spot counts (spots per well, spw) are mean values of duplicate wells and expressed as spot-forming unit per 2.5x10^5^ PBMCs (spots/2.5x10^5^ PBMCs), per 10,000 CD3^+^ T cells (spots/1.0x10^4^ CD3^+^), and per µl blood (spots/µl blood). Spots/1.0x10^4^ CD3^+^ were calculated considering the frequencies of CD3^+^ T cells within PBMCs determined by flow cytometry and the spw of the respective antigens. Spots/µl blood were calculated with regard to the number of CD45^+^ lymphocytes/µl blood in whole blood determined by flow cytometry. The positive threshold was set at ≥3spw or >2xNC. Donors were divided into four groups: high responders (HR, ≥50spw or 47spw + 2xNC), intermediate responders (IR, ≥10spw or 7spw + 2xNC), low responders (LR, ≥3spw or 2xNC) and non-responders (NR, <3spw or 2xNC). The average value obtained in NC was 0.215spw (range 0-5spw). Only seropositive donors (if applicable) were included in analysis of T-cell responses.

**Supplementary Tables**

**Table S1: Flow cytometry panel for analysis of whole blood samples.** List of antibodies that were used for staining of whole blood samples.

| **Clone** | **Specificity** | **Fluorochrome** | **Manufacturer** |
| --- | --- | --- | --- |
| SK7 | CD3 | FITC | BD Biosciences |
| SK3 | CD4 | PerCP | BD Biosciences |
| NCAM16.2 | CD56 | PE | BD Biosciences |
| 2D1 | CD45 | APC-H7 | BD Biosciences |
| SK1 | CD8 | APC | BD Biosciences |
| MφP9 | CD14 | BV510 | BD Biosciences |
| HI100 | CD45RA | BV605 | BioLegend |
| DREG-56 | CD62L | BV421 | BioLegend |
| 11F2 | γδ TCR | PE-Cy7 | BD Biosciences |
| HIB19 | CD19 | AF-700 | BD Biosciences |

**Table S2: Flow cytometry panel for analysis of PBMCs.** List of antibodies that were used for staining of PBMCs.

| **Clone** | **Specificity** | **Fluorochrome** | **Manufacturer** |
| --- | --- | --- | --- |
| SK7 | CD3 | FITC | BD Biosciences |
| SK3 | CD4 | PerCP | BD Biosciences |
| 2D1 | CD45 | APC-H7 | BD Biosciences |
| SK1 | CD8 | APC | BD Biosciences |
| HI100 | CD45RA | BV605 | BioLegend |
| DREG-56 | CD62L | BV421 | BioLegend |

**Table S3: Peptide pools.** List of peptide pools (pp), consisting mainly of 15-mer sequences with 11 amino acid overlap, that were used in the study.

| **Virus** | **Peptide pool** | **Manufacturer** |
| --- | --- | --- |
| CMV | PepTivator CMV_pp65 | Miltenyi Biotec |
|  | PepTivator CMV_IE1 | Miltenyi Biotec |
| EBV | PepTivator EBV_EBNA1 | Miltenyi Biotec |
|  | PepTivator EBV_Consensus* | Miltenyi Biotec |
|  | PepTivator EBV_LMP2a | Miltenyi Biotec |
|  | PepTivator EBV_BZLF1 | Miltenyi Biotec |
| HHV6 | PepMix HHV6 (U54) | JPT Peptide Technologies |
|  | PepMix HHV6 (U90) | JPT Peptide Technologies |
| HSV | PepTivator HHV1 (gD) | Miltenyi Biotec |
|  | PepMix HSV2 (VP22) | JPT Peptide Technologies |
|  | PepMix HSV2 (gD) | JPT Peptide Technologies |
| VZV | PepMix VZV (gE) | JPT Peptide Technologies |
|  | PepMix VZV (IE62) Vial 1 | JPT Peptide Technologies |
|  | PepMix VZV (IE62) Vial 2 | JPT Peptide Technologies |
| ADV | PepTivator ADV5_Hexon | Miltenyi Biotec |
|  | PepTivator ADV5_Penton | Miltenyi Biotec |
|  | PepTivator ADV_Select* | Miltenyi Biotec |
| BKV | PepTivator BKV_VP1 | Miltenyi Biotec |
|  | PepTivator BKV_LT | Miltenyi Biotec |
| JCV | PepTivator JCV_VP1 | Miltenyi Biotec |
|  | PepTivator JCV_LT | Miltenyi Biotec |
| RSV | PepTivator RSV Nucleoprotein | Miltenyi Biotec |
| IAV | PepTivator Influenza A MP1 | Miltenyi Biotec |

*selected epitopes. CMV cytomegalovirus, EBV Epstein-Barr virus, HHV6 human herpesvirus 6, HSV herpes simplex virus, VZV varicella-zoster virus, ADV adenovirus, BKV BK polyomavirus, JCV JC polyomavirus, RSV respiratory syncytial virus, IAV influenza A virus

**Table S4: Relation and total number of positive serological tests (I).** Number of donors with positive serology for indicated combinations of viruses.

|  | **CMV** | **EBV** | **HSV** | **VZV** | **ADV** | **RSV** | **IAV** |
| --- | --- | --- | --- | --- | --- | --- | --- |
| **CMV**  recomLine | **66** | 63 | 50 | 61 | 60 | 49 | 63 |
| **EBV**  recomLine | 63 | **134** | 95 | 127 | 122 | 111 | 126 |
| **HSV**  recomLine | 50 | 95 | **98** | 91 | 87 | 78 | 90 |
| **VZV**  NovaLisa | 61 | 127 | 91 | **144** | 132 | 121 | 136 |
| **ADV**  NovaLisa | 60 | 122 | 87 | 132 | **138** | 115 | 129 |
| **RSV**  NovaLisa | 49 | 111 | 78 | 121 | 115 | **127** | 120 |
| **IAV**  NovaLisa | 63 | 126 | 90 | 136 | 129 | 120 | **142** |

CMV cytomegalovirus, EBV Epstein-Barr virus, HHV6 human herpesvirus 6, HSV herpes simplex virus, VZV varicella-zoster virus, ADV adenovirus, BKV BK polyomavirus, JCV JC polyomavirus, RSV respiratory syncytial virus, IAV influenza A virus

**Table S5: Relation and total number of positive serological tests (II).** Number and frequency of seropositive donors, their mean age and number of positive donors of the respective group.

| **serology** | **total**  n positive (%) | **total**  age (years) | **m≤40 years**  n positive (%) | **m>40 years**  n positive (%) | **f≤40 years**  n positive (%) | **f>40 years**  n positive (%) |
| --- | --- | --- | --- | --- | --- | --- |
| 7 | 27 (17.9%) | 45.8 | 6 (15.0%) | 10 (21.3%) | 5 (16.7%) | 6 (17.6%) |
| 6 | 59 (39.1%) | 40.8 | 18 (45.0%) | 18 (38.3%) | 11 (36.7%) | 12 (35.3%) |
| 5 | 46 (30.5%) | 42.8 | 9 (22.5%) | 13 (27.7%) | 10 (33.3%) | 14 (41.2%) |
| 4 | 19 (12.6%) | 37.1 | 7 (17.5%) | 6 (12.8%) | 4 (13.3%) | 2 (5.88%) |
| 3 | 0 (0.00%) | n.a. | 0 (0.00%) | 0 (0.00%) | 0 (0.00%) | 0 (0.00%) |
| 2 | 0 (0.00%) | n.a. | 0 (0.00%) | 0 (0.00%) | 0 (0.00%) | 0 (0.00%) |
| 1 | 0 (0.00%) | n.a. | 0 (0.00%) | 0 (0.00%) | 0 (0.00%) | 0 (0.00%) |
| 0 | 0 (0.00%) | n.a. | 0 (0.00%) | 0 (0.00%) | 0 (0.00%) | 0 (0.00%) |

n.a. not applicable

**Table S6A: Reference values based on serological results and frequencies of virus-specific T-cells in healthy male donors (m≤40 years, n=40).**

| **Virus** | **Antigen** | **Min** | **25% Percentile** | **Median** | **75% Percentile** | **Max** | **Mean** | **SD** | **Lower 95% CI**  **of mean** | **Upper 95% CI**  **of mean** |
| --- | --- | --- | --- | --- | --- | --- | --- | --- | --- | --- |
| seropositive out of total  n (%) |  |  |  |  |  |  |  |  |  |  |
| **CMV**  18 (45.0) | pp65 | 0.25 | 5.25 | 12.01 | 24.66 | 40.56 | 14.85 | 11.28 | 9.24 | 20.46 |
|  | IE-1 | 0.07 | 1.05 | 2.21 | 13.43 | 41.96 | 8.76 | 12.99 | 2.31 | 15.22 |
| **EBV**  34 (85.0) | EBNA-1 | 0.00 | 0.00 | 0.24 | 0.91 | 17.30 | 1.53 | 3.58 | 0.28 | 2.78 |
|  | Consensus | 0.42 | 2.50 | 5.49 | 14.35 | 42.77 | 10.32 | 11.71 | 6.24 | 14.41 |
|  | LMP2a | 0.00 | 0.06 | 0.33 | 1.03 | 6.09 | 0.87 | 1.43 | 0.37 | 1.37 |
|  | BZLF-1 | 0.00 | 0.24 | 1.79 | 3.96 | 52.25 | 4.84 | 9.89 | 1.39 | 8.29 |
| **HHV6** | U54 | 0.00 | 0.16 | 0.34 | 0.72 | 4.05 | 0.54 | 0.74 | 0.31 | 0.78 |
|  | U90 | 0.05 | 0.84 | 1.81 | 3.13 | 8.33 | 2.27 | 2.08 | 1.60 | 2.94 |
| **HSV**  20 (50.0) | HSV1_gD | 0.00 | 0.09 | 0.27 | 1.03 | 2.61 | 0.61 | 0.70 | 0.28 | 0.94 |
|  | HSV2_VP22 | 0.00 | 0.17 | 1.28 | 3.10 | 35.12 | 3.84 | 7.94 | 0.12 | 7.55 |
|  | HSV2_gD | 0.05 | 0.16 | 0.50 | 1.20 | 5.32 | 0.90 | 1.24 | 0.32 | 1.48 |
| **VZV**  39 (97.5) | gE | 0.00 | 0.35 | 0.66 | 1.42 | 3.17 | 0.92 | 0.78 | 0.67 | 1.17 |
|  | IE62_1 | 0.00 | 0.15 | 0.41 | 1.00 | 7.54 | 1.02 | 1.64 | 0.49 | 1.56 |
|  | IE62_2 | 0.04 | 0.25 | 0.71 | 1.31 | 4.97 | 1.11 | 1.18 | 0.72 | 1.49 |
| **ADV**  37 (92.5) | Hexon | 0.07 | 0.83 | 1.69 | 3.92 | 31.23 | 3.61 | 5.56 | 1.75 | 5.46 |
|  | Penton | 0.04 | 0.32 | 0.74 | 2.02 | 18.40 | 2.36 | 4.30 | 0.93 | 3.79 |
|  | Select | 0.04 | 0.38 | 0.95 | 1.98 | 9.23 | 1.65 | 2.07 | 0.96 | 2.34 |
| **BKV** | VP1 | 0.00 | 0.00 | 0.28 | 0.91 | 4.81 | 0.71 | 1.10 | 0.36 | 1.06 |
|  | LT | 0.00 | 0.04 | 0.23 | 0.46 | 3.29 | 0.39 | 0.57 | 0.20 | 0.57 |
| **JCV** | VP1 | 0.00 | 0.00 | 0.08 | 0.32 | 1.45 | 0.25 | 0.37 | 0.13 | 0.36 |
|  | LT | 0.00 | 0.00 | 0.10 | 0.30 | 1.28 | 0.24 | 0.32 | 0.14 | 0.34 |
| **RSV**  34 (85.0) | NP | 0.00 | 0.05 | 0.51 | 0.86 | 3.23 | 0.70 | 0.84 | 0.41 | 1.00 |
| **IAV**  40 (100.0) | MP1 | 0.00 | 0.07 | 0.32 | 0.94 | 10.20 | 1.13 | 2.31 | 0.39 | 1.87 |

CMV cytomegalovirus, EBV Epstein-Barr virus, HHV6 human herpesvirus 6, HSV herpes simplex virus, VZV varicella-zoster virus, ADV adenovirus, BKV BK polyomavirus, JCV JC polyomavirus, RSV respiratory syncytial virus, IAV influenza A virus

**Table S6B: Reference values based on serological results and frequencies of virus-specific T-cells in healthy male donors (m>40 years, n=47).**

| **Virus** | **Antigen** | **Min** | **25% Percentile** | **Median** | **75% Percentile** | **Max** | **Mean** | **SD** | **Lower 95% CI**  **of mean** | **Upper 95% CI**  **of mean** |
| --- | --- | --- | --- | --- | --- | --- | --- | --- | --- | --- |
| seropositive out of total  n (%) |  |  |  |  |  |  |  |  |  |  |
| **CMV**  15 (31.9) | pp65 | 1.74 | 7.35 | 18.62 | 29.91 | 46.42 | 18.99 | 13.48 | 11.52 | 26.45 |
|  | IE-1 | 0.21 | 2.88 | 4.99 | 13.65 | 67.26 | 13.44 | 19.51 | 2.63 | 24.24 |
| **EBV**  42 (89.4) | EBNA-1 | 0.00 | 0.00 | 0.27 | 1.13 | 35.90 | 1.47 | 5.50 | -0.24 | 3.19 |
|  | Consensus | 0.00 | 3.84 | 7.49 | 22.15 | 86.61 | 15.70 | 19.29 | 9.69 | 21.71 |
|  | LMP2a | 0.00 | 0.31 | 1.19 | 2.59 | 41.83 | 3.60 | 7.97 | 1.11 | 6.08 |
|  | BZLF-1 | 0.00 | 0.21 | 1.59 | 7.89 | 63.34 | 8.43 | 16.03 | 3.43 | 13.42 |
| **HHV6** | U54 | 0.00 | 0.15 | 0.45 | 0.96 | 19.25 | 1.15 | 2.93 | 0.29 | 2.01 |
|  | U90 | 0.00 | 1.04 | 1.99 | 3.43 | 71.34 | 4.95 | 11.21 | 1.66 | 8.25 |
| **HSV**  37 (78.7) | HSV1_gD | 0.00 | 0.07 | 0.31 | 1.18 | 5.32 | 0.87 | 1.28 | 0.44 | 1.30 |
|  | HSV2_VP22 | 0.08 | 0.77 | 2.13 | 4.00 | 17.98 | 3.26 | 3.81 | 1.99 | 4.53 |
|  | HSV2_gD | 0.00 | 0.19 | 0.64 | 1.33 | 14.78 | 1.27 | 2.48 | 0.44 | 2.10 |
| **VZV**  44 (93.6) | gE | 0.00 | 0.20 | 0.63 | 1.99 | 16.58 | 1.78 | 3.16 | 0.82 | 2.74 |
|  | IE62_1 | 0.00 | 0.12 | 0.32 | 0.89 | 4.32 | 0.68 | 0.91 | 0.40 | 0.95 |
|  | IE62_2 | 0.00 | 0.19 | 0.56 | 0.92 | 9.43 | 0.83 | 1.44 | 0.39 | 1.27 |
| **ADV**  45 (95.7) | Hexon | 0.00 | 0.39 | 0.83 | 2.55 | 14.88 | 2.21 | 3.15 | 1.26 | 3.15 |
|  | Penton | 0.00 | 0.09 | 0.32 | 0.74 | 5.37 | 0.74 | 1.18 | 0.39 | 1.09 |
|  | Select | 0.00 | 0.28 | 0.66 | 1.56 | 11.77 | 1.52 | 2.41 | 0.80 | 2.25 |
| **BKV** | VP1 | 0.00 | 0.10 | 0.30 | 0.65 | 12.62 | 0.84 | 1.96 | 0.26 | 1.41 |
|  | LT | 0.00 | 0.00 | 0.24 | 0.55 | 6.14 | 0.58 | 1.19 | 0.23 | 0.93 |
| **JCV** | VP1 | 0.00 | 0.00 | 0.16 | 0.44 | 4.86 | 0.44 | 0.87 | 0.18 | 0.69 |
|  | LT | 0.00 | 0.00 | 0.18 | 0.42 | 3.04 | 0.42 | 0.69 | 0.22 | 0.62 |
| **RSV**  41 (87.2) | NP | 0.00 | 0.11 | 0.30 | 0.89 | 5.69 | 0.78 | 1.23 | 0.39 | 1.17 |
| **IAV**  43 (91.5) | MP1 | 0.00 | 0.14 | 0.54 | 1.76 | 5.65 | 1.24 | 1.62 | 0.74 | 1.74 |

CMV cytomegalovirus, EBV Epstein-Barr virus, HHV6 human herpesvirus 6, HSV herpes simplex virus, VZV varicella-zoster virus, ADV adenovirus, BKV BK polyomavirus, JCV JC polyomavirus, RSV respiratory syncytial virus, IAV influenza A virus

**Table S6C: Reference values based on serological results and frequencies of virus-specific T-cells in healthy female donors (f≤40 years, n=30).**

| **Virus** | **Antigen** | **Min** | **25% Percentile** | **Median** | **75% Percentile** | **Max** | **Mean** | **SD** | **Lower 95% CI**  **of mean** | **Upper 95% CI**  **of mean** |
| --- | --- | --- | --- | --- | --- | --- | --- | --- | --- | --- |
| seropositive out of total  n (%) |  |  |  |  |  |  |  |  |  |  |
| **CMV**  16 (53.3) | pp65 | 0.58 | 3.33 | 5.53 | 12.33 | 17.46 | 7.09 | 5.26 | 4.28 | 9.89 |
|  | IE-1 | 0.00 | 0.26 | 4.91 | 9.62 | 40.80 | 7.70 | 10.87 | 1.91 | 13.50 |
| **EBV**  25 (83.3) | EBNA-1 | 0.00 | 0.05 | 0.42 | 0.87 | 6.00 | 0.73 | 1.25 | 0.21 | 1.24 |
|  | Consensus | 0.06 | 2.14 | 5.92 | 11.76 | 15.36 | 6.65 | 4.85 | 4.65 | 8.65 |
|  | LMP2a | 0.00 | 0.10 | 0.46 | 1.12 | 5.09 | 0.82 | 1.18 | 0.34 | 1.31 |
|  | BZLF-1 | 0.00 | 0.49 | 1.34 | 3.47 | 18.78 | 3.03 | 4.54 | 1.16 | 4.91 |
| **HHV6** | U54 | 0.00 | 0.21 | 0.45 | 0.73 | 10.36 | 1.03 | 2.22 | 0.19 | 1.86 |
|  | U90 | 0.13 | 1.06 | 1.98 | 3.15 | 15.36 | 2.75 | 3.07 | 1.60 | 3.89 |
| **HSV**  16 (53.3) | HSV1_gD | 0.00 | 0.20 | 0.42 | 0.76 | 2.36 | 0.71 | 0.83 | 0.26 | 1.15 |
|  | HSV2_VP22 | 0.07 | 0.40 | 1.20 | 2.39 | 5.03 | 1.57 | 1.39 | 0.83 | 2.31 |
|  | HSV2_gD | 0.06 | 0.26 | 0.35 | 2.05 | 3.52 | 1.00 | 1.11 | 0.41 | 1.59 |
| **VZV**  28 (93.3) | gE | 0.11 | 0.37 | 0.60 | 0.92 | 2.28 | 0.72 | 0.54 | 0.51 | 0.93 |
|  | IE62_1 | 0.12 | 0.26 | 0.42 | 0.83 | 7.06 | 1.08 | 1.64 | 0.45 | 1.72 |
|  | IE62_2 | 0.00 | 0.29 | 0.86 | 2.32 | 7.08 | 1.41 | 1.53 | 0.82 | 2.01 |
| **ADV**  27 (90.0) | Hexon | 0.53 | 1.54 | 2.24 | 4.35 | 9.17 | 3.16 | 2.40 | 2.21 | 4.11 |
|  | Penton | 0.07 | 0.29 | 1.08 | 1.65 | 7.36 | 1.63 | 1.95 | 0.86 | 2.40 |
|  | Select | 0.12 | 0.52 | 1.41 | 2.57 | 4.27 | 1.59 | 1.23 | 1.11 | 2.08 |
| **BKV** | VP1 | 0.00 | 0.14 | 0.34 | 0.66 | 2.74 | 0.56 | 0.65 | 0.32 | 0.80 |
|  | LT | 0.00 | 0.07 | 0.15 | 0.36 | 2.06 | 0.34 | 0.49 | 0.15 | 0.52 |
| **JCV** | VP1 | 0.00 | 0.04 | 0.11 | 0.43 | 0.98 | 0.23 | 0.25 | 0.14 | 0.32 |
|  | LT | 0.00 | 0.07 | 0.13 | 0.29 | 2.23 | 0.30 | 0.47 | 0.13 | 0.48 |
| **RSV**  27 (90.0) | NP | 0.00 | 0.13 | 0.42 | 0.97 | 2.15 | 0.59 | 0.63 | 0.34 | 0.84 |
| **IAV**  28 (93.3) | MP1 | 0.00 | 0.30 | 0.45 | 1.06 | 2.02 | 0.65 | 0.53 | 0.45 | 0.86 |

CMV cytomegalovirus, EBV Epstein-Barr virus, HHV6 human herpesvirus 6, HSV herpes simplex virus, VZV varicella-zoster virus, ADV adenovirus, BKV BK polyomavirus, JCV JC polyomavirus, RSV respiratory syncytial virus, IAV influenza A virus

**Table S6D: Reference values based on serological results and frequencies of virus-specific T-cells in healthy female donors (f>40 years, n=34).**

| **Virus** | **Antigen** | **Min** | **25% Percentile** | **Median** | **75% Percentile** | **Max** | **Mean** | **SD** | **Lower 95% CI**  **of mean** | **Upper 95% CI**  **of mean** |
| --- | --- | --- | --- | --- | --- | --- | --- | --- | --- | --- |
| seropositive out of total  n (%) |  |  |  |  |  |  |  |  |  |  |
| **CMV**  17 (50.0) | pp65 | 1.74 | 3.81 | 9.45 | 18.23 | 38.90 | 12.00 | 10.69 | 6.50 | 17.49 |
|  | IE-1 | 0.00 | 0.45 | 5.53 | 12.23 | 83.76 | 11.69 | 21.38 | 0.70 | 22.68 |
| **EBV**  33 (97.1) | EBNA-1 | 0.00 | 0.08 | 0.28 | 1.56 | 8.51 | 1.32 | 2.24 | 0.52 | 2.11 |
|  | Consensus | 0.70 | 2.66 | 8.82 | 17.01 | 61.83 | 11.82 | 12.72 | 7.31 | 16.33 |
|  | LMP2a | 0.00 | 0.05 | 0.68 | 2.14 | 11.77 | 1.34 | 2.24 | 0.55 | 2.13 |
|  | BZLF-1 | 0.00 | 0.07 | 0.64 | 4.21 | 29.31 | 3.85 | 6.51 | 1.54 | 6.15 |
| **HHV6** | U54 | 0.00 | 0.07 | 0.34 | 0.54 | 4.71 | 0.49 | 0.81 | 0.21 | 0.78 |
|  | U90 | 0.25 | 0.85 | 1.44 | 4.04 | 6.68 | 2.29 | 1.86 | 1.64 | 2.93 |
| **HSV**  25 (73.5) | HSV1_gD | 0.00 | 0.04 | 0.27 | 0.79 | 8.47 | 0.90 | 1.77 | 0.17 | 1.64 |
|  | HSV2_VP22 | 0.00 | 0.19 | 1.24 | 1.98 | 14.72 | 2.15 | 3.19 | 0.83 | 3.46 |
|  | HSV2_gD | 0.00 | 0.10 | 0.36 | 1.10 | 13.07 | 1.32 | 2.72 | 0.19 | 2.44 |
| **VZV**  33 (97.1) | gE | 0.00 | 0.17 | 0.51 | 1.36 | 8.12 | 1.16 | 1.78 | 0.53 | 1.79 |
|  | IE62_1 | 0.00 | 0.23 | 0.44 | 1.05 | 4.93 | 0.88 | 1.14 | 0.47 | 1.28 |
|  | IE62_2 | 0.00 | 0.15 | 0.45 | 1.19 | 7.84 | 1.08 | 1.78 | 0.45 | 1.72 |
| **ADV**  28 (82.4) | Hexon | 0.08 | 0.29 | 0.72 | 2.13 | 4.64 | 1.22 | 1.25 | 0.74 | 1.71 |
|  | Penton | 0.03 | 0.14 | 0.28 | 1.21 | 3.57 | 0.82 | 1.05 | 0.41 | 1.22 |
|  | Select | 0.00 | 0.22 | 0.82 | 1.53 | 5.02 | 1.09 | 1.15 | 0.65 | 1.54 |
| **BKV** | VP1 | 0.00 | 0.02 | 0.17 | 0.53 | 3.06 | 0.36 | 0.58 | 0.16 | 0.57 |
|  | LT | 0.00 | 0.00 | 0.11 | 0.36 | 3.12 | 0.42 | 0.79 | 0.14 | 0.70 |
| **JCV** | VP1 | 0.00 | 0.00 | 0.10 | 0.24 | 1.19 | 0.19 | 0.27 | 0.09 | 0.28 |
|  | LT | 0.00 | 0.05 | 0.15 | 0.30 | 1.98 | 0.29 | 0.43 | 0.14 | 0.44 |
| **RSV**  25 (73.5) | NP | 0.00 | 0.07 | 0.36 | 0.83 | 2.03 | 0.53 | 0.60 | 0.28 | 0.77 |
| **IAV**  31 (91.2) | MP1 | 0.00 | 0.08 | 0.39 | 1.20 | 2.85 | 0.62 | 0.71 | 0.37 | 0.88 |

CMV cytomegalovirus, EBV Epstein-Barr virus, HHV6 human herpesvirus 6, HSV herpes simplex virus, VZV varicella-zoster virus, ADV adenovirus, BKV BK polyomavirus, JCV JC polyomavirus, RSV respiratory syncytial virus, IAV influenza A virus

**Table S7: T-cell phenotype distribution in relation to antiviral T-cell responses in seropositive healthy donors.**

| **Virus** | **Antigen** | **Pheno-**  **type** | **CD3^+^** | | | | **CD4^+^** | | | | **CD8^+^** | | | |
| --- | --- | --- | --- | --- | --- | --- | --- | --- | --- | --- | --- | --- | --- | --- |
|  |  |  | **HR** | **IR** | **LR** | **NR** | **HR** | **IR** | **LR** | **NR** | **HR** | **IR** | **LR** | **NR** |
| **CMV** | pp65 | T_N_ | 33.8 | 38.0 | 38.6 | *40.4** | 32.8 | 36.9 | 41.0 | *38.4** | 37.0 | 44.1 | 39.1 | *47.9** |
|  |  | T_CM_ | 28.3 | 27.7 | 26.2 | *31.5** | 40.1 | 36.9 | 37.6 | *41.4** | 11.1 | 12.4 | 7.7 | *14.3** |
|  |  | T_EM_ | 21.7 | 20.1 | 23.5 | *20.7** | 23.2 | 20.7 | 19.4 | *18.7** | 21.1 | 16.8 | 25.3 | *22.7** |
|  |  | T_EMRA_ | 16.2 | 14.2 | 11.7 | *7.3** | 3.7 | 5.5 | 2.0 | *1.4** | 30.9 | 26.7 | 28.0 | *15.1** |
|  | IE-1 | T_N_ | 35.2 | 32.8 | 35.2 | 37.8 | 33.9 | 32.5 | 35.9 | 36.3 | 38.5 | 35.3 | 40.2 | 44.1 |
|  |  | T_CM_ | 29.5 | 24.7 | 24.7 | 32.2 | 42.7 | 33.9 | 32.9 | 42.9 | 11.4 | 9.9 | 12.5 | 12.3 |
|  |  | T_EM_ | 21.0 | 21.7 | 23.9 | 20.6 | 21.1 | 26.3 | 22.4 | 19.4 | 20.4 | 18.7 | 22.1 | 21.5 |
|  |  | T_EMRA_ | 14.3 | 20.8 | 16.2 | 9.3 | 2.3 | 7.3 | 6.3 | 1.5 | 29.7 | 36.1 | 25.2 | 22.2 |
| **EBV** | EBNA-1 | T_N_ | 31.0 | 37.5 | 38.3 | 38.3 | 28.5 | 35.6 | 38.8 | 36.6 | 37.3 | 44.7 | 42.2 | 43.5 |
|  |  | T_CM_ | 40.7 | 30.4 | 29.4 | 28.8 | 51.5 | 41.5 | 39.7 | 39.6 | 22.3 | 12.6 | 13.2 | 11.8 |
|  |  | T_EM_ | 21.5 | 22.4 | 20.2 | 20.7 | 19.0 | 19.7 | 19.0 | 20.8 | 24.5 | 24.7 | 20.1 | 20.9 |
|  |  | T_EMRA_ | 6.8 | 9.7 | 12.1 | 12.2 | 1.0 | 3.2 | 2.2 | 3.0 | 15.9 | 18.0 | 24.5 | 23.8 |
|  | Consensus | T_N_ | 37.1 | 37.5 | 40.7 | 44.5 | 35.2 | 37.8 | 36.7 | 43.0 | 42.5 | 41.3 | 50.8 | 54.3 |
|  |  | T_CM_ | 31.3 | 28.5 | 28.5 | 25.6 | 43.0 | 36.5 | 43.6 | 34.4 | 13.9 | 12.5 | 8.8 | 9.6 |
|  |  | T_EM_ | 21.1 | 21.0 | 19.0 | 20.3 | 20.1 | 20.5 | 19.0 | 18.3 | 21.5 | 22.7 | 18.2 | 19.5 |
|  |  | T_EMRA_ | 10.5 | 13.0 | 11.7 | 9.6 | 1.7 | 4.8 | 0.7 | 4.3 | 22.1 | 23.5 | 22.3 | 16.6 |
|  | LMP2A | T_N_ | 28.1 | 39.8 | 37.0 | 38.3 | 25.4 | 38.0 | 35.7 | 37.6 | 32.4 | 47.0 | 41.1 | 43.2 |
|  |  | T_CM_ | 34.3 | 31.4 | 29.5 | 28.9 | 45.0 | 42.4 | 39.5 | 39.8 | 18.4 | 13.3 | 12.0 | 12.6 |
|  |  | T_EM_ | 27.0 | 20.1 | 19.3 | 21.3 | 27.8 | 18.1 | 20.8 | 19.5 | 25.5 | 21.3 | 19.3 | 22.4 |
|  |  | T_EMRA_ | 10.6 | 8.7 | 14.2 | 11.6 | 1.8 | 1.5 | 3.9 | 2.9 | 23.8 | 18.5 | 27.5 | 21.8 |
|  | BZLF-1 | T_N_ | 35.0 | 37.0 | 41.9 | 38.6 | 33.5 | 35.3 | 42.2 | 37.2 | 39.1 | 44.0 | 43.9 | 44.7 |
|  |  | T_CM_ | 30.2 | 32.4 | 29.6 | 28.2 | 43.1 | 43.6 | 37.6 | 37.7 | 13.2 | 13.8 | 13.9 | 12.0 |
|  |  | T_EM_ | 22.3 | 21.5 | 20.5 | 19.5 | 22.0 | 19.2 | 17.4 | 20.3 | 22.2 | 23.6 | 23.0 | 18.8 |
|  |  | T_EMRA_ | 12.5 | 9.1 | 7.9 | 13.7 | 1.4 | 1.8 | 2.0 | 4.8 | 25.6 | 18.6 | 19.1 | 24.5 |
| **HHV6** | U54 | T_N_ | 35.2 | 35.3 | 39.4 | 37.8 | 33.2 | 33.3 | 38.3 | 36.0 | 38.9 | 43.5 | 44.5 | 43.7 |
|  |  | T_CM_ | 33.7 | 34.8 | 29.9 | 28.7 | 47.7 | 45.9 | 39.7 | 39.4 | 13.5 | 16.7 | 12.9 | 12.0 |
|  |  | T_EM_ | 19.1 | 20.6 | 21.2 | 21.1 | 17.1 | 19.6 | 20.0 | 21.0 | 22.0 | 20.3 | 21.9 | 21.8 |
|  |  | T_EMRA_ | 12.0 | 9.3 | 9.6 | 12.3 | 2.1 | 1.2 | 1.9 | 3.5 | 25.6 | 19.4 | 20.7 | 22.4 |
|  | U90 | T_N_ | 36.2 | 38.2 | 38.2 | 39.6 | 35.9 | 36.7 | 36.1 | 37.7 | 37.6 | 43.7 | 45.9 | 49.4 |
|  |  | T_CM_ | 27.9 | 30.8 | 28.5 | 31.9 | 39.1 | 40.9 | 39.5 | 42.7 | 13.5 | 13.4 | 11.2 | 13.8 |
|  |  | T_EM_ | 20.8 | 20.7 | 22.5 | 19.3 | 21.3 | 19.9 | 21.6 | 18.2 | 20.8 | 22.6 | 20.8 | 18.8 |
|  |  | T_EMRA_ | 15.2 | 10.3 | 10.7 | 9.2 | 3.7 | 2.4 | 2.9 | 1.4 | 28.2 | 20.3 | 22.0 | 17.9 |
| **HSV** | HSV1  gD | T_N_ | 32.5 | 36.9 | 35.9 | 36.6 | 35.4 | 36.5 | 33.9 | 35.4 | 31.9 | 40.7 | 41.8 | 42.3 |
|  |  | T_CM_ | 26.0 | 28.0 | 33.4 | 29.6 | 39.6 | 38.1 | 45.7 | 39.0 | 10.9 | 12.7 | 14.2 | 13.1 |
|  |  | T_EM_ | 19.8 | 22.1 | 20.9 | 21.4 | 22.3 | 20.9 | 19.3 | 21.7 | 16.9 | 22.2 | 21.9 | 20.9 |
|  |  | T_EMRA_ | 21.5 | 12.9 | 9.8 | 12.4 | 2.8 | 4.5 | 1.1 | 3.6 | 40.3 | 24.4 | 22.1 | 23.7 |
|  | HSV2  VP22 | T_N_ | 36.3 | 35.9 | 38.3 | 35.9 | 34.9 | 34.4 | 37.3 | 35.1 | 41.3 | 40.8 | 47.1 | 39.0 |
|  |  | T_CM_ | 28.7 | 32.1 | 29.4 | 28.6 | 40.9 | 42.9 | 40.4 | 36.9 | 11.4 | 14.6 | 11.3 | 13.5 |
|  |  | T_EM_ | 21.2 | 22.1 | 21.1 | 20.3 | 20.8 | 20.7 | 19.9 | 22.1 | 19.1 | 23.2 | 18.9 | 21.5 |
|  |  | T_EMRA_ | 21.2 | 10.0 | 11.2 | 15.2 | 3.5 | 2.0 | 2.4 | 5.4 | 28.3 | 21.4 | 22.7 | 26.0 |
|  | HSV2  gD | T_N_ | 28.3 | 36.2 | 37.1 | 36.6 | 24.9 | 36.2 | 36.0 | 34.8 | 35.7 | 40.1 | 43.1 | 42.1 |
|  |  | T_CM_ | 33.3 | 28.4 | 31.1 | 30.7 | 44.6 | 39.3 | 42.4 | 40.3 | 14.1 | 11.7 | 13.9 | 13.8 |
|  |  | T_EM_ | 26.5 | 21.5 | 21.9 | 20.6 | 28.9 | 20.9 | 19.5 | 21.2 | 19.0 | 21.1 | 23.5 | 20.4 |
|  |  | T_EMRA_ | 11.9 | 13.9 | 10.0 | 12.1 | 1.6 | 3.6 | 2.0 | 3.5 | 31.2 | 27.0 | 19.6 | 23.7 |
| **VZV** | gE | T_N_ | 47.6 | 34.9 | 39.7 | 38.2 | 53.1 | 32.5 | 37.7 | 38.2 | 36.6 | 40.8 | 47.3 | 42.7 |
|  |  | T_CM_ | 16.6 | 31.3 | 30.9 | 28.8 | 22.9 | 41.1 | 42.0 | 39.2 | 7.7 | 13.2 | 13.5 | 12.7 |
|  |  | T_EM_ | 15.4 | 22.7 | 20.8 | 20.2 | 13.6 | 22.7 | 18.8 | 20.2 | 18.4 | 23.7 | 22.3 | 19.8 |
|  |  | T_EMRA_ | 20.4 | 11.0 | 8.5 | 12.8 | 10.4 | 3.5 | 1.6 | 2.4 | 37.3 | 22.3 | 16.8 | 24.9 |
|  | IE62_1 | T_N_ | 42.7 | 36.6 | 37.7 | 39.0 | 46.0 | 34.4 | 37.2 | 37.7 | 37.2 | 43.6 | 41.1 | 45.6 |
|  |  | T_CM_ | 25.3 | 31.4 | 28.0 | 30.6 | 41.3 | 43.0 | 37.9 | 40.5 | 5.1 | 13.9 | 11.0 | 14.0 |
|  |  | T_EM_ | 10.1 | 22.1 | 21.1 | 20.6 | 12.1 | 20.7 | 20.7 | 19.8 | 6.7 | 23.2 | 22.7 | 20.7 |
|  |  | T_EMRA_ | 21.9 | 9.8 | 13.2 | 9.8 | 0.7 | 1.8 | 4.2 | 2.0 | 51.1 | 19.3 | 25.2 | 19.7 |
|  | IE62_2 | T_N_ | 33.6 | 38.8 | 35.5 | 39.9 | 28.0 | 36.6 | 35.2 | 38.9 | 47.4 | 44.3 | 39.4 | 46.7 |
|  |  | T_CM_ | 33.9 | 30.4 | 30.1 | 29.4 | 44.6 | 39.7 | 42.1 | 39.1 | 15.1 | 13.0 | 12.7 | 13.2 |
|  |  | T_EM_ | 27.5 | 20.1 | 22.0 | 20.5 | 26.5 | 19.9 | 20.9 | 19.5 | 27.6 | 22.0 | 23.7 | 19.7 |
|  |  | T_EMRA_ | 5.0 | 10.7 | 12.4 | 10.2 | 0.8 | 3.7 | 1.8 | 2.6 | 9.9 | 20.7 | 24.2 | 20.4 |
| **ADV** | Hexon | T_N_ | 35.1 | 39.3 | 38.3 | 38.3 | 37.6 | 37.7 | 36.9 | 36.2 | 35.0 | 45.4 | 44.4 | 44.1 |
|  |  | T_CM_ | 29.1 | 30.4 | 30.7 | 29.7 | 41.7 | 42.4 | 42.4 | 39.0 | 12.5 | 11.9 | 11.8 | 14.2 |
|  |  | T_EM_ | 23.1 | 20.0 | 21.1 | 20.9 | 19.2 | 18.4 | 19.5 | 21.7 | 28.1 | 21.7 | 22.7 | 20.6 |
|  |  | T_EMRA_ | 12.7 | 10.3 | 9.9 | 11.0 | 1.5 | 1.5 | 1.2 | 3.1 | 24.4 | 20.9 | 21.1 | 21.2 |
|  | Penton | T_N_ | 38.1 | 38.3 | 37.6 | 40.2 | 36.3 | 36.3 | 36.7 | 39.2 | 43.7 | 44.5 | 41.0 | 47.7 |
|  |  | T_CM_ | 32.0 | 30.8 | 30.2 | 26.6 | 44.0 | 42.5 | 39.1 | 37.4 | 13.1 | 13.0 | 13.3 | 11.5 |
|  |  | T_EM_ | 22.0 | 20.9 | 20.2 | 20.5 | 18.6 | 19.8 | 21.1 | 20.4 | 25.8 | 21.8 | 22.2 | 17.8 |
|  |  | T_EMRA_ | 8.0 | 10.0 | 12.1 | 12.7 | 1.0 | 1.5 | 3.1 | 3.0 | 17.3 | 20.6 | 23.6 | 23.0 |
|  | Select | T_N_ | 39.8 | 39.5 | 36.9 | 38.2 | 37.6 | 38.8 | 35.5 | 35.8 | 44.0 | 44.2 | 43.3 | 44.7 |
|  |  | T_CM_ | 29.0 | 30.6 | 32.1 | 27.7 | 39.9 | 42.4 | 41.9 | 38.7 | 12.3 | 12.7 | 14.1 | 11.8 |
|  |  | T_EM_ | 20.6 | 19.4 | 21.9 | 21.5 | 20.3 | 17.2 | 20.8 | 22.7 | 20.8 | 21.7 | 23.5 | 20.6 |
|  |  | T_EMRA_ | 10.6 | 10.4 | 9.1 | 12.6 | 2.3 | 1.6 | 1.8 | 2.8 | 23.0 | 21.4 | 19.0 | 22.9 |
| **BKV** | VP1 | T_N_ | 37.7 | 38.0 | 38.4 | 37.9 | 36.5 | 37.5 | 35.3 | 36.8 | 42.8 | 41.3 | 45.5 | 43.8 |
|  |  | T_CM_ | 34.8 | 24.7 | 32.2 | 30.1 | 44.5 | 36.2 | 44.4 | 39.6 | 10.4 | 10.0 | 14.1 | 13.1 |
|  |  | T_EM_ | 18.5 | 22.0 | 20.6 | 21.1 | 17.4 | 21.7 | 19.1 | 20.7 | 20.3 | 22.0 | 21.8 | 21.5 |
|  |  | T_EMRA_ | 9.0 | 15.3 | 8.8 | 10.9 | 1.6 | 4.6 | 1.1 | 2.8 | 26.5 | 26.7 | 18.5 | 21.6 |
|  | LT | T_N_ | - | 40.4 | 35.9 | 38.5 | - | 39.8 | 35.5 | 36.5 | - | 44.3 | 39.9 | 45.2 |
|  |  | T_CM_ | - | 24.0 | 30.7 | 30.5 | - | 32.9 | 42.1 | 40.9 | - | 8.6 | 14.5 | 13.0 |
|  |  | T_EM_ | - | 20.9 | 20.9 | 21.1 | - | 21.3 | 20.1 | 20.3 | - | 18.8 | 22.7 | 21.7 |
|  |  | T_EMRA_ | - | 14.7 | 12.5 | 9.9 | - | 6.0 | 2.3 | 2.2 | - | 28.3 | 22.9 | 20.2 |
| **JCV** | VP1 | T_N_ | - | 30.2 | 38.5 | 38.4 | - | 31.4 | 36.9 | 36.7 | - | 31.0 | 43.9 | 44.6 |
|  |  | T_CM_ | - | 25.3 | 30.6 | 30.1 | - | 34.2 | 42.6 | 40.3 | - | 9.5 | 14.0 | 12.9 |
|  |  | T_EM_ | - | 23.0 | 21.4 | 20.9 | - | 24.8 | 19.1 | 20.4 | - | 20.2 | 23.4 | 21.3 |
|  |  | T_EMRA_ | - | 21.5 | 9.5 | 10.7 | - | 9.6 | 1.4 | 2.5 | - | 39.3 | 18.7 | 21.3 |
|  | LT | T_N_ | - | 33.6 | 38.0 | 38.6 | - | 32.6 | 37.9 | 36.7 | - | 37.7 | 40.9 | 45.3 |
|  |  | T_CM_ | - | 29.2 | 28.5 | 30.4 | - | 39.2 | 40.1 | 40.7 | - | 12.1 | 13.6 | 12.9 |
|  |  | T_EM_ | - | 23.9 | 20.7 | 20.8 | - | 22.5 | 19.9 | 20.2 | - | 24.3 | 20.5 | 21.6 |
|  |  | T_EMRA_ | - | 13.3 | 12.7 | 10.3 | - | 5.7 | 2.2 | 2.3 | - | 25.9 | 25.0 | 20.2 |
| **RSV** | NP | T_N_ | - | 38.3 | 39.4 | 38.4 | - | 35.6 | 38.4 | 36.9 | - | 45.8 | 44.3 | 45.0 |
|  |  | T_CM_ | - | 34.9 | 30.2 | 28.6 | - | 46.0 | 41.3 | 39.0 | - | 15.9 | 14.2 | 11.9 |
|  |  | T_EM_ | - | 21.0 | 19.5 | 21.8 | - | 17.6 | 18.1 | 21.6 | - | 25.4 | 20.1 | 21.3 |
|  |  | T_EMRA_ | - | 5.9 | 10.9 | 11.2 | - | 0.8 | 2.2 | 2.3 | - | 12.9 | 21.4 | 21.8 |
| **IAV** | MP1 | T_N_ | 30.0 | 38.4 | 37.1 | 39.2 | 29.2 | 36.6 | 35.7 | 37.7 | 32.3 | 45.1 | 41.7 | 45.6 |
|  |  | T_CM_ | 33.2 | 30.6 | 33.0 | 26.7 | 48.1 | 42.1 | 43.4 | 36.4 | 14.9 | 13.0 | 15.5 | 10.7 |
|  |  | T_EM_ | 24.6 | 21.0 | 20.7 | 20.9 | 21.7 | 19.5 | 19.1 | 21.9 | 28.5 | 21.5 | 23.1 | 20.1 |
|  |  | T_EMRA_ | 12.1 | 10.0 | 9.2 | 13.2 | 1.1 | 1.8 | 1.7 | 4.0 | 24.3 | 20.5 | 19.7 | 23.6 |

CMV cytomegalovirus, EBV Epstein-Barr virus, HHV6 human herpesvirus 6, HSV herpes simplex virus, VZV varicella-zoster virus, ADV adenovirus, BKV BK polyomavirus, JCV JC polyomavirus, RSV respiratory syncytial virus, IAV influenza A virus. T_N_ naïve T cell (CD45RA^+^CD62L^+^), T_CM_ Central Memory T cells (CD45RA^-^CD62L^+^), T_EM_ Effector Memory T cells (CD45RA^-^CD62L^-^), T_EMRA_ Effector Memory T cell re-expressing CD45RA (CD45RA^+^CD62L^-^). HR high responder (≥50spw or 47spw + 2 x negative control (NC), IR intermediate responder (≥10spw or 7spw + 2xNC), LR low responder (≥3spw or 2xNC), NR non-responder (<3spw or 2xNC).

*) No NR for CMV_pp65 observed. Values shown were obtained from CMV-seronegative donors.

**SUPPLEMENTARY FIGURES**


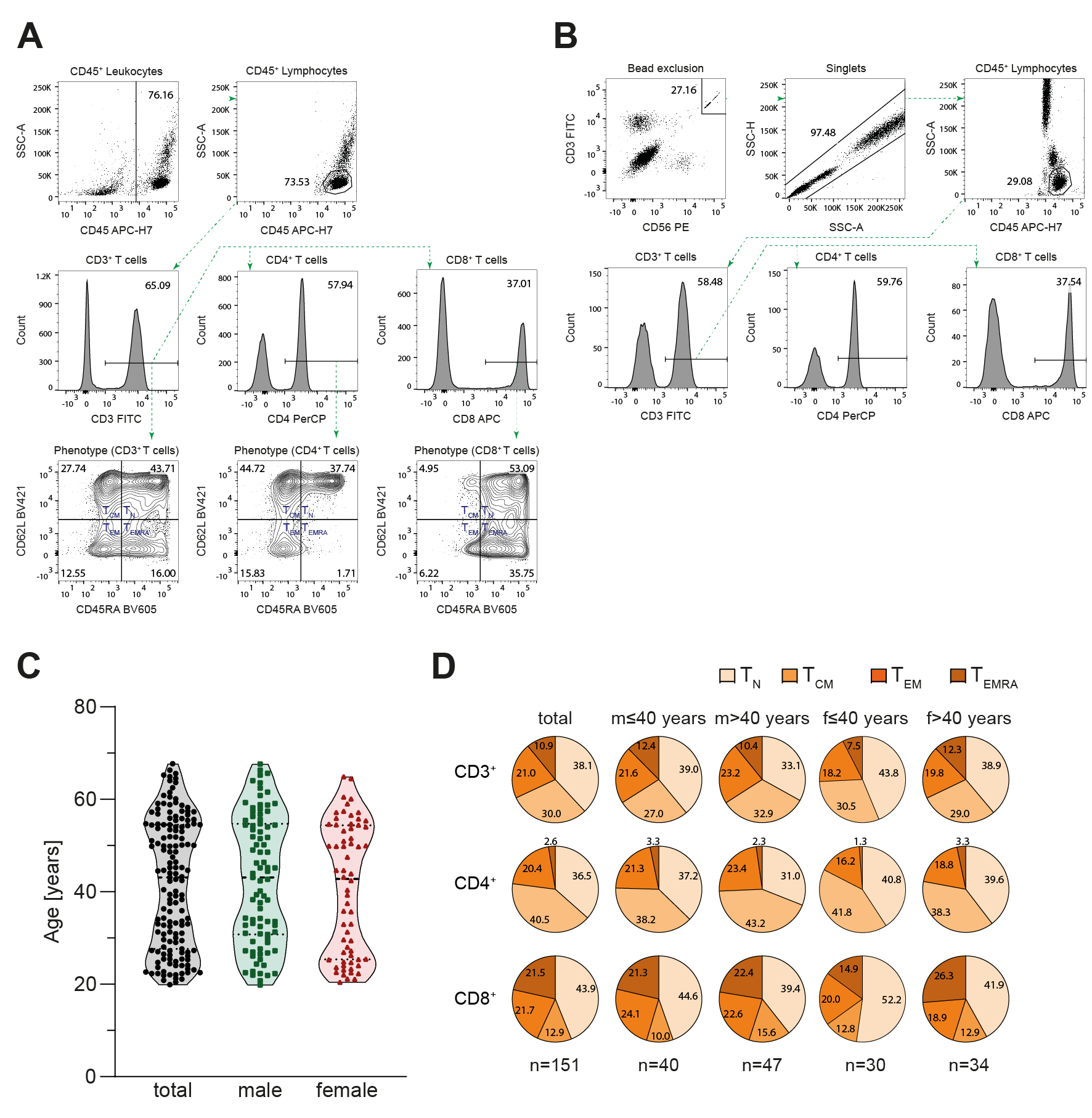


**Figure S1. Donor cohort.** (**A**) Gating strategy: CD3^+^ T cell frequencies within PBMCs and T-cell memory phenotypes. (**B**) Gating strategy: CD3^+^ T cell counts per µl blood. (**C**) Age distribution among donor cohort in total (n=151) and among male (n=87) and female donors (n=64). (**D**) T-cell phenotypes of donor cohort in total (n=151) and among males until the age of 40 (m≤40 years, n=40), males above the age of 40 (m>40 years, n=47), females until the age of 40 (f≤40 years, n=30), and (4) females above the age of 40 (f>40 years, n=34). T_N_: naïve T cell (CD45RA^+^CD62L^+^), T_CM_: Central Memory T cells (CD45RA^-^CD62L^+^), T_EM_: Effector Memory T cells (CD45RA^-^CD62L^-^), T_EMRA_: Effector Memory T cell re-expressing CD45RA (CD45RA^+^CD62L^-^).


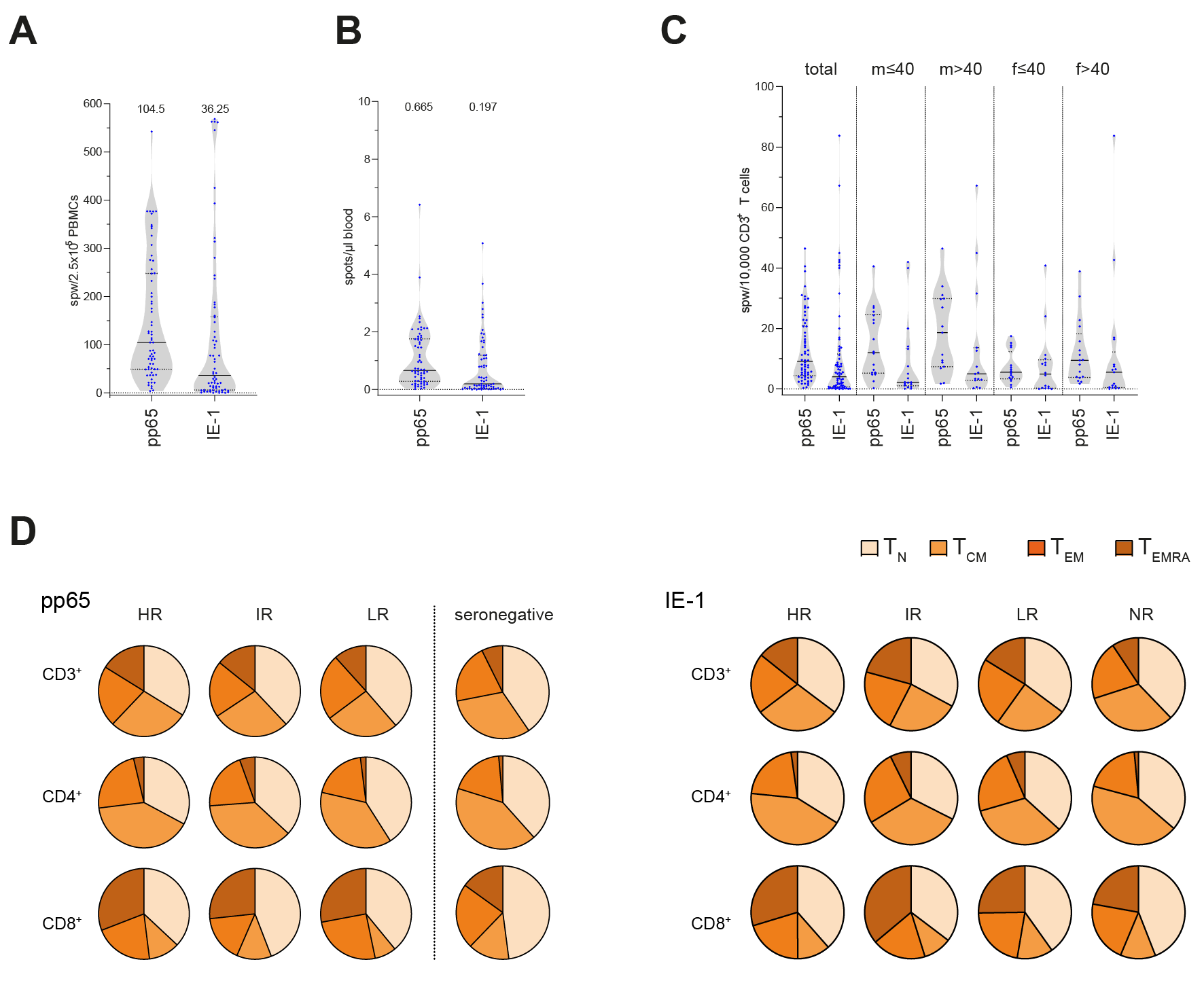


**Figure S2. Detection of Cytomegalovirus (CMV)-specific memory T cells.** CMV_pp65- and CMV_IE-1-specific T cells in CMV-seropositive healthy donors (n=66) were detected by ELISpot and analyzed with respect to frequencies and memory subsets, as well as donor age and gender. (**A**) Frequencies of antigen-specific T cells among CMV-seropositive donors expressed as spw/2.5x10^5^ PBMCs. (**B**) Frequencies of antigen-specific T cells among CMV-seropositive donors expressed as spots/µl blood. (**A,B**) The number above each data set indicates the median. (**C**) Distribution of T-cell responses against CMV-specific peptide pools among CMV-seropositive donors in the whole group and donor groups divided by age and gender. (**D**) T-cell phenotypes for each CMV-seropositive responder group and for CMV-seronegative donors. T_N_: naïve T cell (CD45RA^+^CD62L^+^), T_CM_: Central Memory T cells (CD45RA^-^CD62L^+^), T_EM_: Effector Memory T cells (CD45RA^-^CD62L^-^), T_EMRA_: Effector Memory T cell re-expressing CD45RA (CD45RA^+^CD62L^-^), HR: High Responder, IR: Intermediate Responder, LR: Low Responder, NR: Non-Responder.


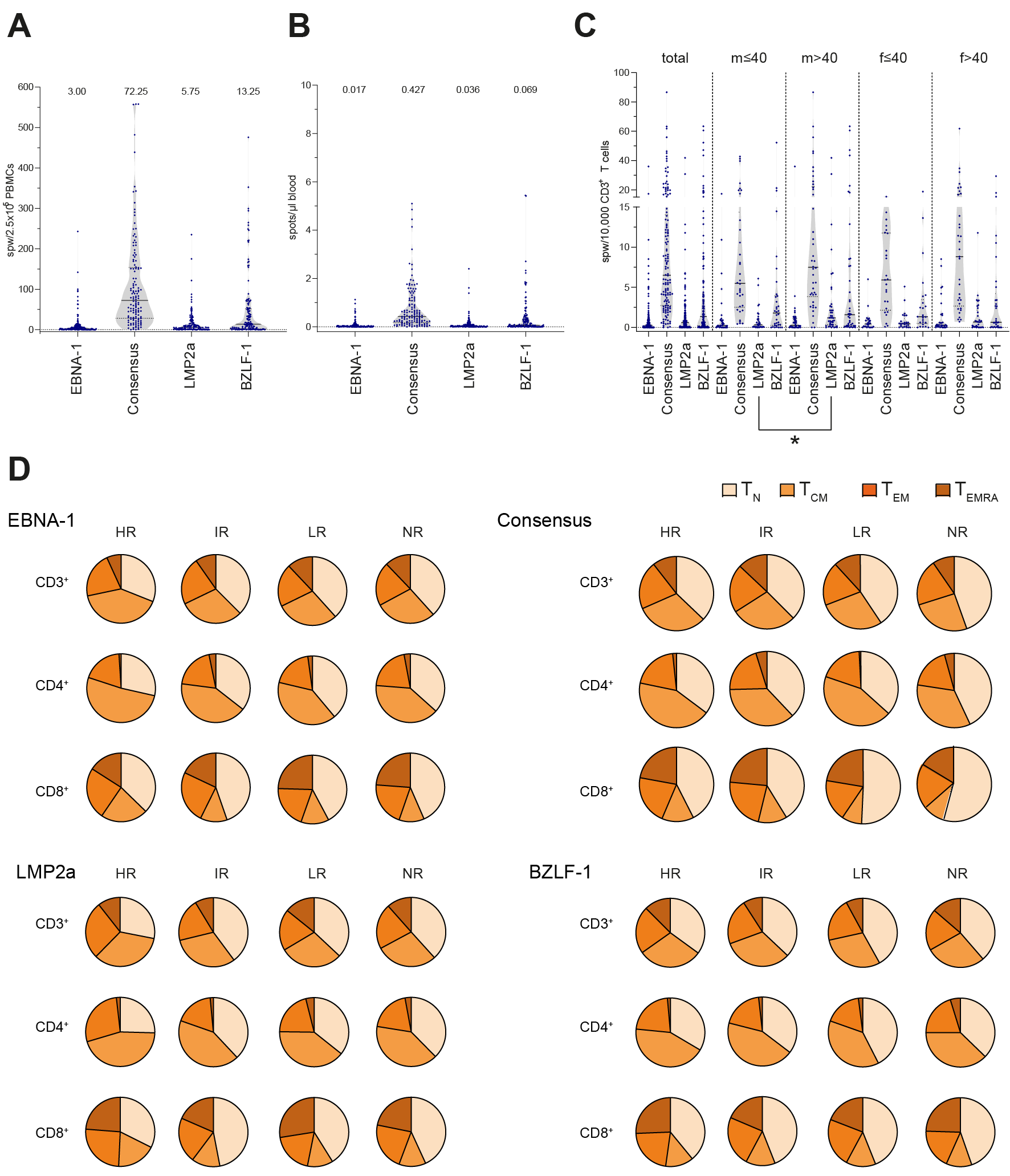


**Figure S3. Detection of Epstein-Barr Virus (EBV)-specific memory T cells.** EBV_EBNA-1-, EBV_Consensus-, EBV_LMP2a- and EBV_BZLF-1-specific T cells in EBV-seropositive healthy donors (n=134) were detected by ELISpot and analyzed with respect to frequencies and memory subsets, as well as donor age and gender. (**A**) Frequencies of antigen-specific T cells among EBV-seropositive donors expressed as spw/2.5x10^5^ PBMCs. (**B**) Frequencies of antigen-specific T cells among EBV-seropositive donors expressed as spots/µl blood. (**A,B**) The number above each data set indicates the median. (**C**) Distribution of T-cell responses against EBV-specific peptide pools among EBV-seropositive donors in the whole group and donor groups divided by age and gender. Asterisk indicates statistically significant difference between gender- and age-related T-cell frequencies (Mann-Whitney). (**D**) T-cell phenotypes for each EBV responder group. T_N_: naïve T cell (CD45RA^+^CD62L^+^), T_CM_: Central Memory T cells (CD45RA^-^CD62L^+^), T_EM_: Effector Memory T cells (CD45RA^-^CD62L^-^), T_EMRA_: Effector Memory T cell re-expressing CD45RA (CD45RA^+^CD62L^-^), HR: High Responder, IR: Intermediate Responder, LR: Low Responder, NR: Non-Responder. * p< 0.05.


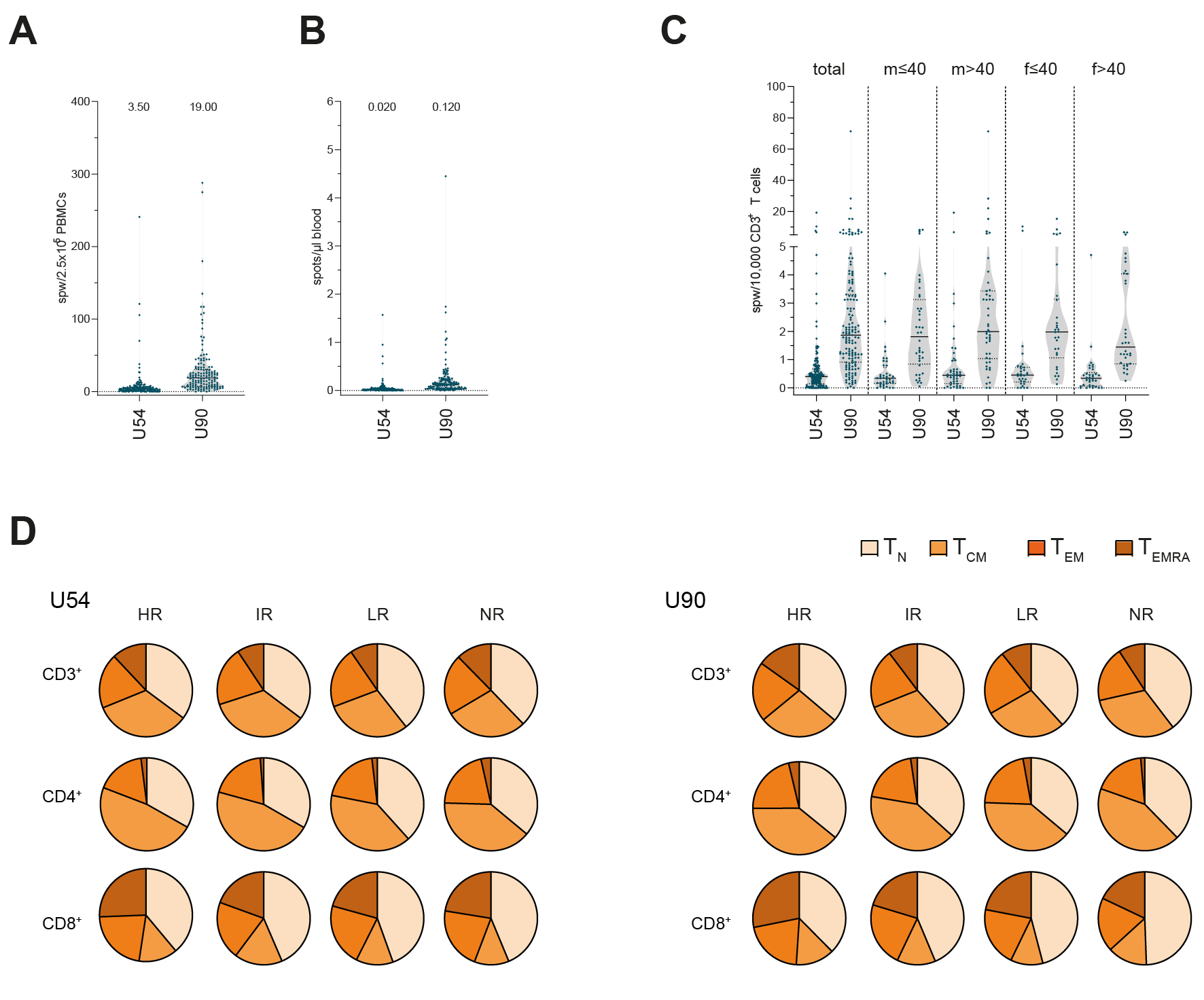


**Figure S4. Detection of Human Herpesvirus 6 (HHV6)-specific memory T cells.** HHV6_U54- and HHV6_U90-specific T cells in healthy donors (n=151) were detected by ELISpot and analyzed with respect to frequencies and memory subsets, as well as donor age and gender. (**A**) Frequencies of antigen-specific T cells expressed as spw/2.5x10^5^ PBMCs. (**B**) Frequencies of antigen-specific T cells expressed as spots/µl blood. (**A,B**) The number above each data set indicates the median. (**C**) Distribution of T-cell responses against HHV6-specific peptide pools in the whole group and donor groups divided by age and gender. (**D**) T-cell phenotypes for each HHV6 responder group. T_N_: naïve T cell (CD45RA^+^CD62L^+^), T_CM_: Central Memory T cells (CD45RA^-^CD62L^+^), T_EM_: Effector Memory T cells (CD45RA^-^CD62L^-^), T_EMRA_: Effector Memory T cell re-expressing CD45RA (CD45RA^+^CD62L^-^), HR: High Responder, IR: Intermediate Responder, LR: Low Responder, NR: Non-Responder.


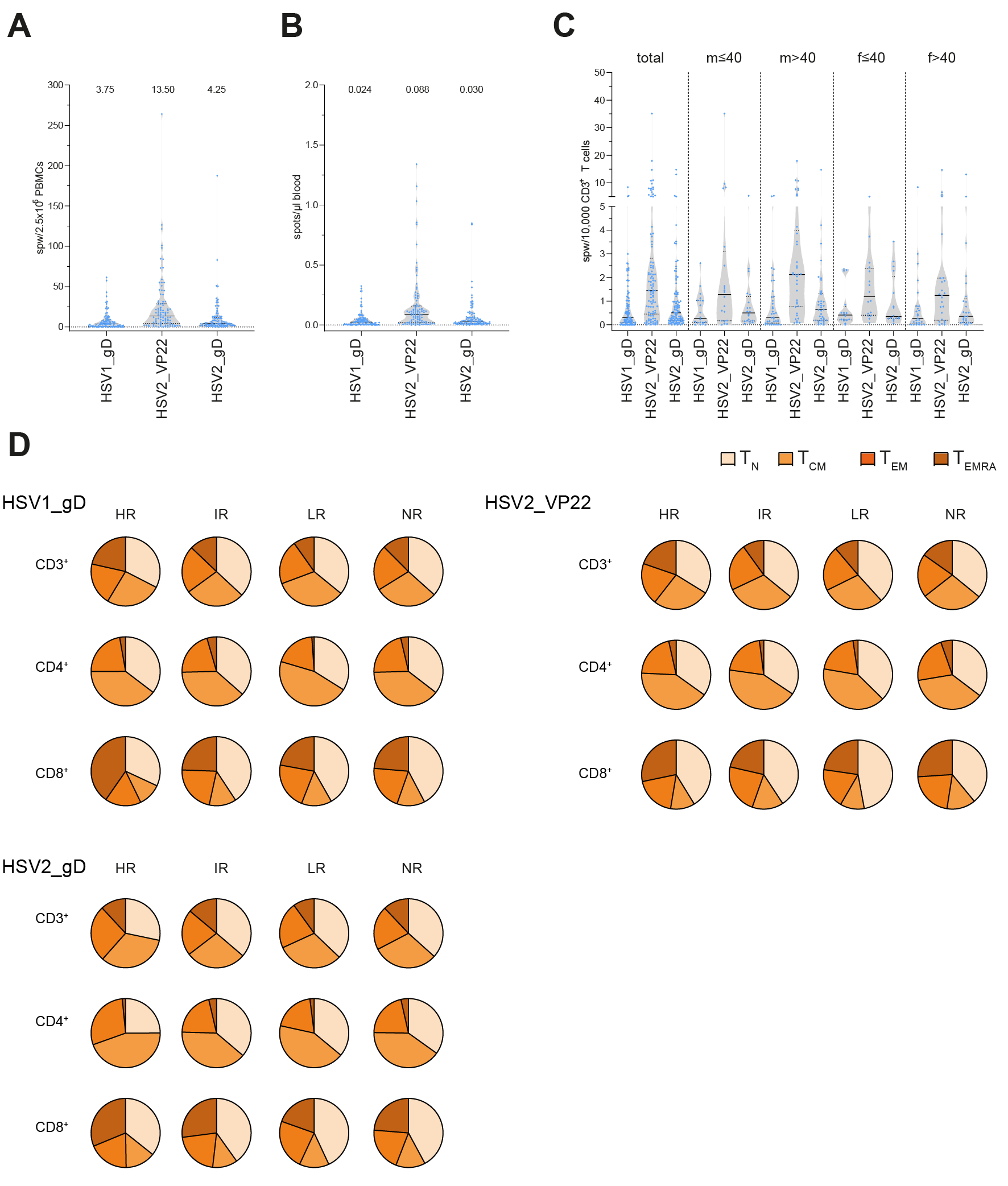


**Figure S5. Detection of Herpes Simplex Virus (HSV)-specific memory T cells.** HSV1_gD-, HSV2_VP22- and HSV2_gD-specific T cells in HSV-seropositive healthy donors (n=98) were detected by ELISpot and analyzed with respect to frequencies and memory subsets, as well as donor age and gender. (**A**) Frequencies of antigen-specific T cells among HSV-seropositive donors expressed as spw/2.5x10^5^ PBMCs. (**B**) Frequencies of antigen-specific T cells among HSV-seropositive donors expressed as spots/µl blood. (**A,B**) The number above each data set indicates the median. (**C**) Distribution of T-cell responses against HSV-specific peptide pools among HSV-seropositive donors in the whole group and donor groups divided by age and gender. (**D**) T-cell phenotypes for each HSV responder group. T_N_: naïve T cell (CD45RA^+^CD62L^+^), T_CM_: Central Memory T cells (CD45RA^-^CD62L^+^), T_EM_: Effector Memory T cells (CD45RA^-^CD62L^-^), T_EMRA_: Effector Memory T cell re-expressing CD45RA (CD45RA^+^CD62L^-^), HR: High Responder, IR: Intermediate Responder, LR: Low Responder, NR: Non-Responder.


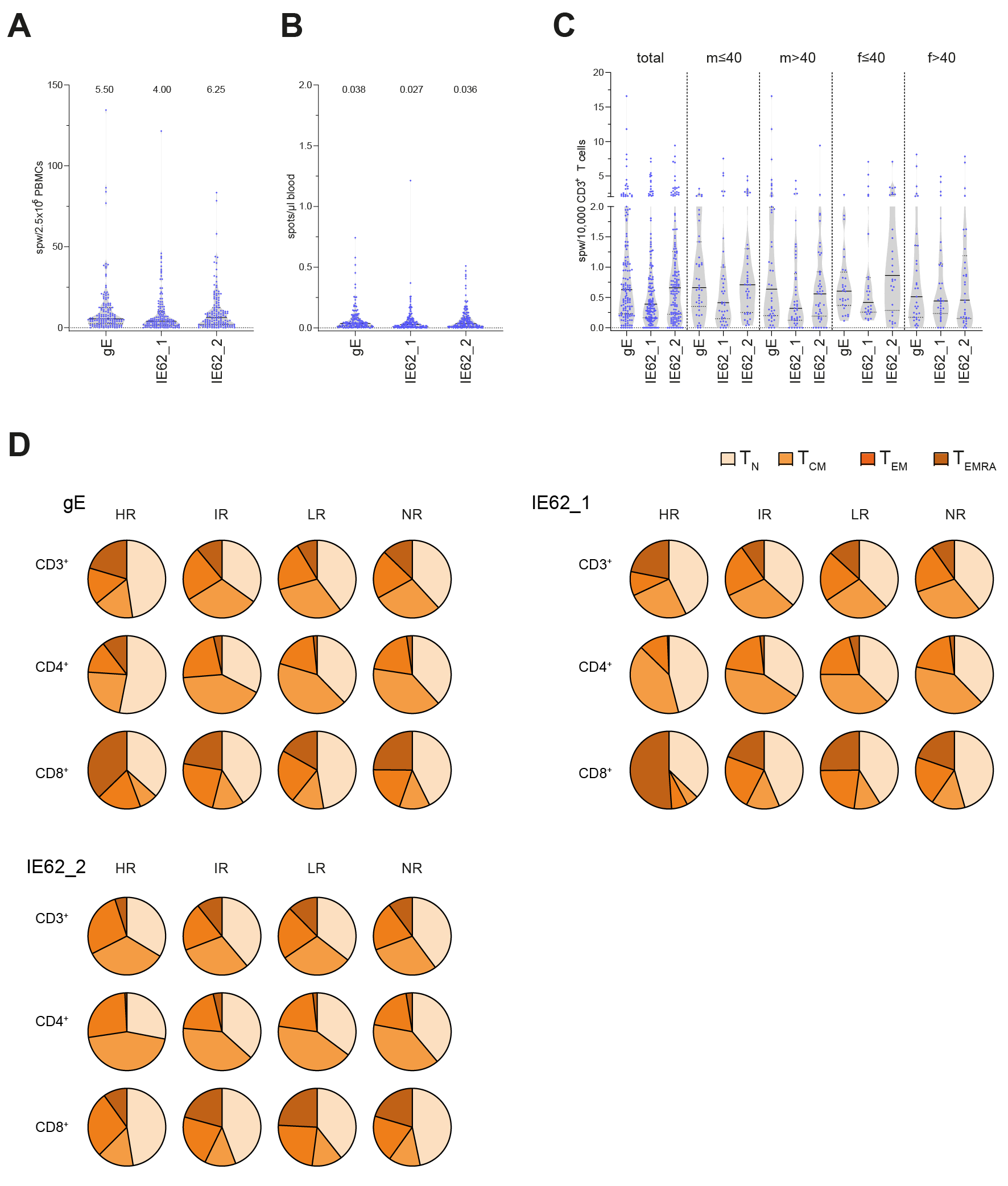


**Figure S6. Detection of Varicella-Zoster Virus (VZV)-specific memory T cells.** VZV_gE-, VZV_IE62_1- and VZV_IE62_2-specific T cells in VZV-seropositive healthy donors (n=144) were detected by ELISpot and analyzed with respect to frequencies and memory subsets, as well as donor age and gender. (**A**) Frequencies of antigen-specific T cells among VZV-seropositive donors expressed as spw/2.5x10^5^ PBMCs. (**B**) Frequencies of antigen-specific T cells among VZV-seropositive donors expressed as spots/µl blood. (**A,B**) The number above each data set indicates the median. (**C**) Distribution of T-cell responses against VZV-specific peptide pools among VZV-seropositive donors in the whole group and donor groups divided by age and gender. (**D**) T-cell phenotypes for each VZV responder group. T_N_: naïve T cell (CD45RA^+^CD62L^+^), T_CM_: Central Memory T cells (CD45RA^-^CD62L^+^), T_EM_: Effector Memory T cells (CD45RA^-^CD62L^-^), T_EMRA_: Effector Memory T cell re-expressing CD45RA (CD45RA^+^CD62L^-^), HR: High Responder, IR: Intermediate Responder, LR: Low Responder, NR: Non-Responder.


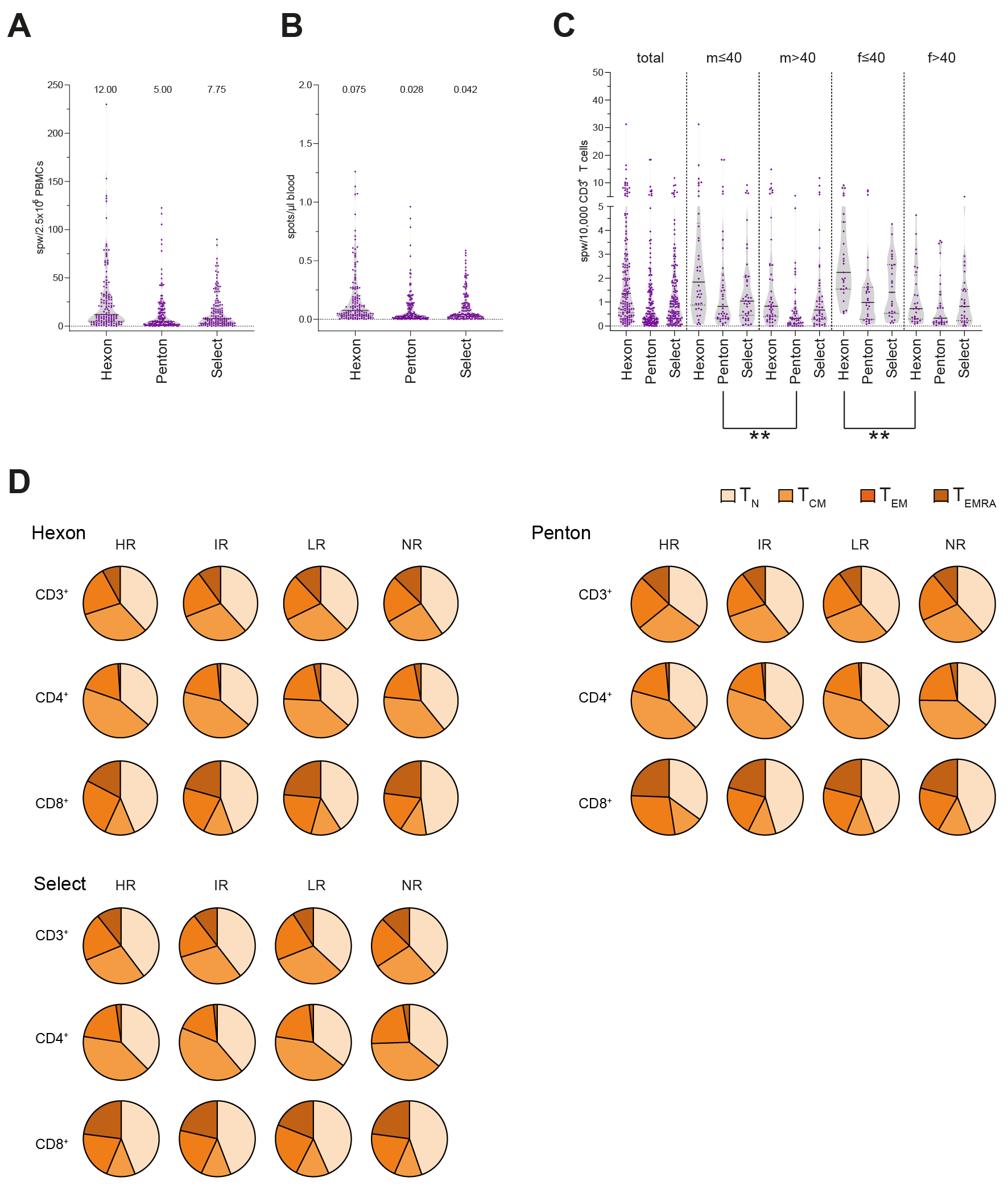


**Figure S7. Detection of Adenovirus (ADV)-specific memory T cells.** ADV_Hexon-, ADV_Penton- and ADV_Select-specific T cells in ADV-seropositive healthy donors (n=138) were detected by ELISpot and analyzed with respect to frequencies and memory subsets, as well as donor age and gender. (**A**) Frequencies of antigen-specific T cells among ADV-seropositive donors expressed as spw/2.5x10^5^ PBMCs. (**B**) Frequencies of antigen-specific T cells among ADV-seropositive donors expressed as spots/µl blood. (**A,B**) The number above each data set indicates the median. (**C**) Distribution of T-cell responses against ADV-specific peptide pools among ADV-seropositive donors in the whole group and donor groups divided by age and gender. Asterisks indicate statistically significant difference between gender- and age-related T-cell frequencies (Mann-Whitney). (**D**) T-cell phenotypes for each ADV responder group. T_N_: naïve T cell (CD45RA^+^CD62L^+^), T_CM_: Central Memory T cells (CD45RA^-^CD62L^+^), T_EM_: Effector Memory T cells (CD45RA^-^CD62L^-^), T_EMRA_: Effector Memory T cell re-expressing CD45RA (CD45RA^+^CD62L^-^), HR: High Responder, IR: Intermediate Responder, LR: Low Responder, NR: Non-Responder. ** p<0.01.


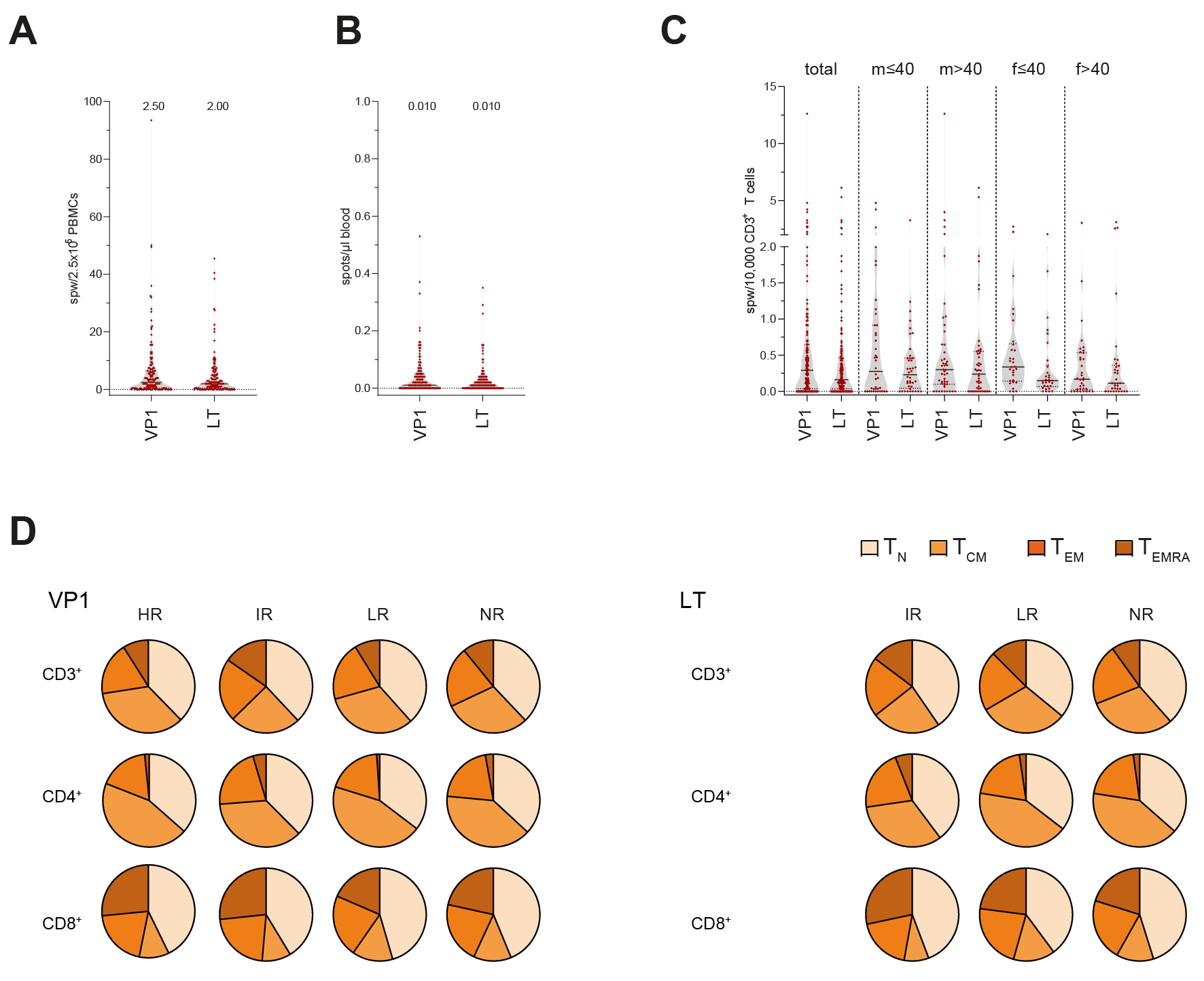


**Figure S8. Detection of BK Polyomavirus (BKV)-specific memory T cells.** BKV_LT- and BKV_VP1-specific T cells in healthy donors (n=151) were detected by ELISpot and analyzed with respect to frequencies and memory subsets, as well as donor age and gender. (**A**) Frequencies of antigen-specific T cells expressed as spw/2.5x10^5^ PBMCs. (**B**) Frequencies of antigen-specific T cells expressed as spots/µl blood. (**A,B**) The number above each data set indicates the median. (**C**) Distribution of T-cell responses against BKV-specific peptide pools in the whole group and donor groups divided by age and gender. (**D**) T-cell phenotypes for each BKV responder group. T_N_: naïve T cell (CD45RA^+^CD62L^+^), T_CM_: Central Memory T cells (CD45RA^-^CD62L^+^), T_EM_: Effector Memory T cells (CD45RA^-^CD62L^-^), T_EMRA_: Effector Memory T cell re-expressing CD45RA (CD45RA^+^CD62L^-^), HR: High Responder, IR: Intermediate Responder, LR: Low Responder, NR: Non-Responder.


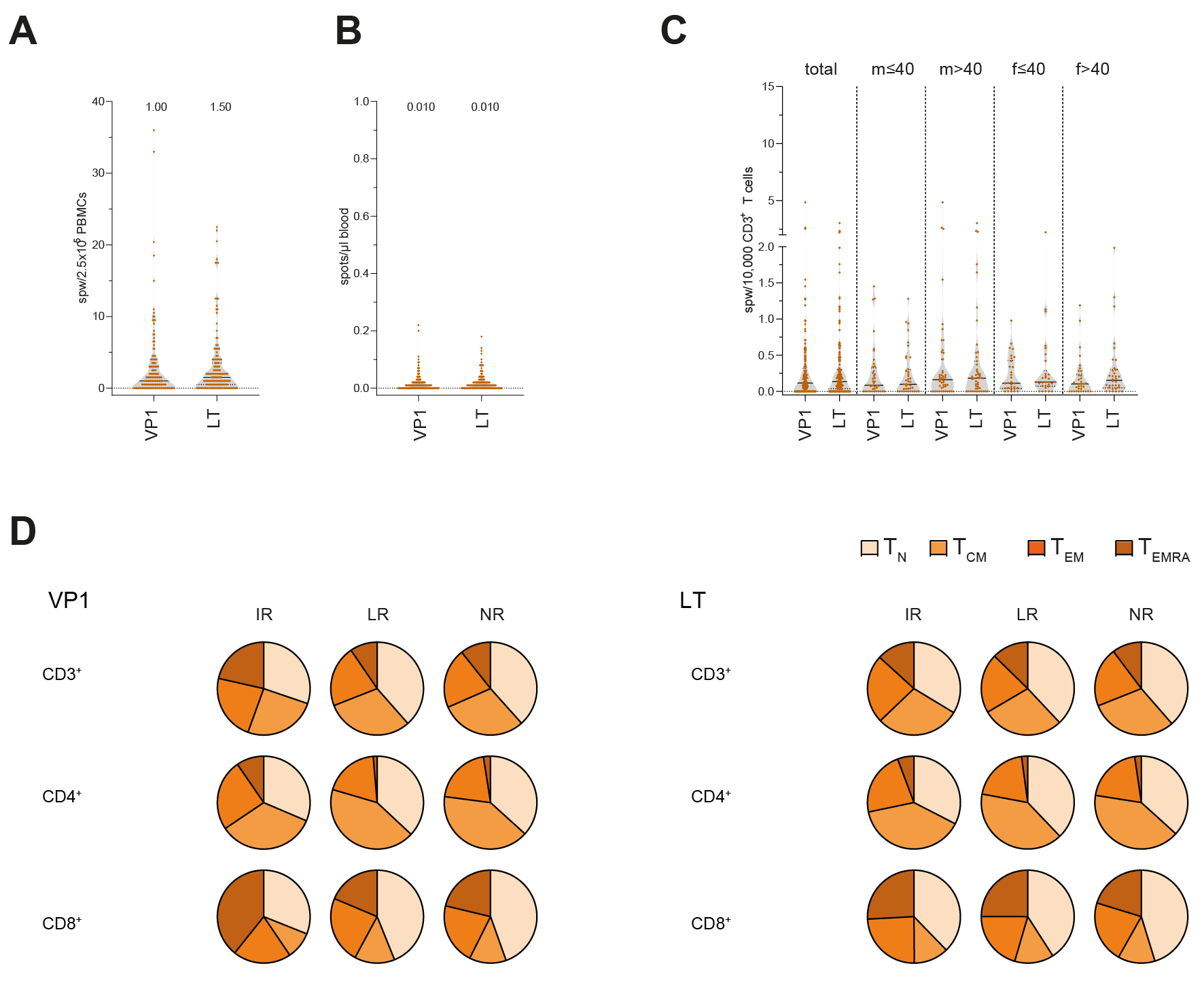


**Figure S9. Detection of JC Polyomavirus (JCV)-specific memory T cells.** JCV_LT- and JCV_VP1-specific T cells in healthy donors (n=151) were detected by ELISpot and analyzed with respect to frequencies and memory subsets, as well as donor age and gender. (**A**) Frequencies of antigen-specific T cells expressed as spw/2.5x10^5^ PBMCs. (**B**) Frequencies of antigen-specific T cells expressed as spots/µl blood. (**A,B**) The number above each data set indicates the median. (**C**) Distribution of T-cell responses against JCV-specific peptide pools in the whole group and donor groups divided by age and gender. (**D**) T-cell phenotypes for each JCV responder group. T_N_: naïve T cell (CD45RA^+^CD62L^+^), T_CM_: Central Memory T cells (CD45RA^-^CD62L^+^), T_EM_: Effector Memory T cells (CD45RA^-^CD62L^-^), T_EMRA_: Effector Memory T cell re-expressing CD45RA (CD45RA^+^CD62L^-^), HR: High Responder, IR: Intermediate Responder, LR: Low Responder, NR: Non-Responder.


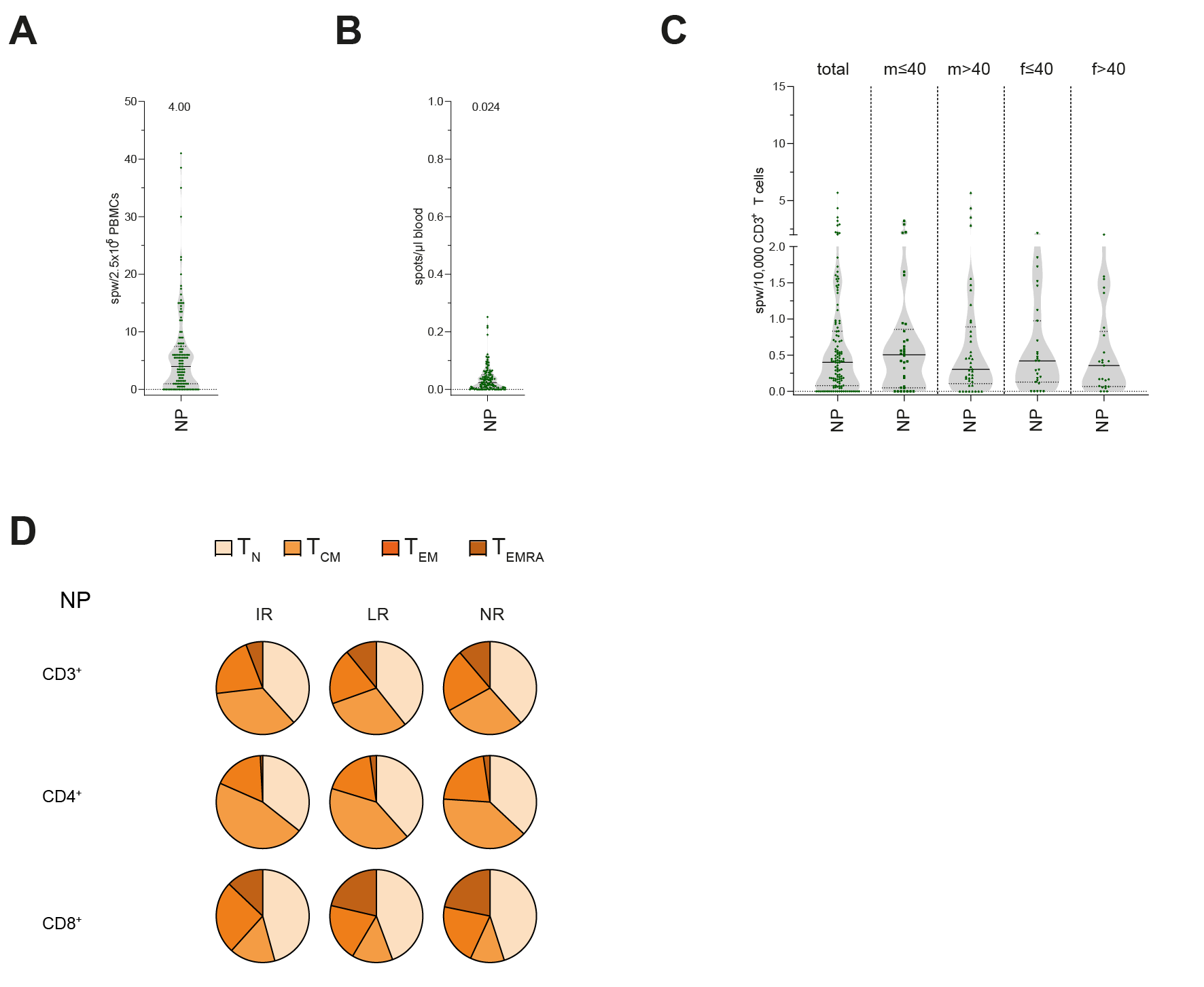


**Figure S10. Detection of Respiratory Syncytial Virus (RSV)-specific memory T cells.** RSV_NP-specific T cells in RSV-seropositive healthy donors (n=127) were detected by ELISpot and analyzed with respect to frequencies and memory subsets, as well as donor age and gender. (**A**) Frequencies of antigen-specific T cells among RSV-seropositive donors expressed as spw/2.5x10^5^ PBMCs. (**B**) Frequencies of antigen-specific T cells among RSV-seropositive donors expressed as spots/µl blood. (**A,B**) The number above each data set indicates the median. (**C**) Distribution of T-cell responses against RSV-specific peptide pool among RSV-seropositive donors in the whole group and donor groups divided by age and gender. (**D**) T-cell phenotypes for each RSV responder group. T_N_: naïve T cell (CD45RA^+^CD62L^+^), T_CM_: Central Memory T cells (CD45RA^-^CD62L^+^), T_EM_: Effector Memory T cells (CD45RA^-^CD62L^-^), T_EMRA_: Effector Memory T cell re-expressing CD45RA (CD45RA^+^CD62L^-^), HR: High Responder, IR: Intermediate Responder, LR: Low Responder, NR: Non-Responder.


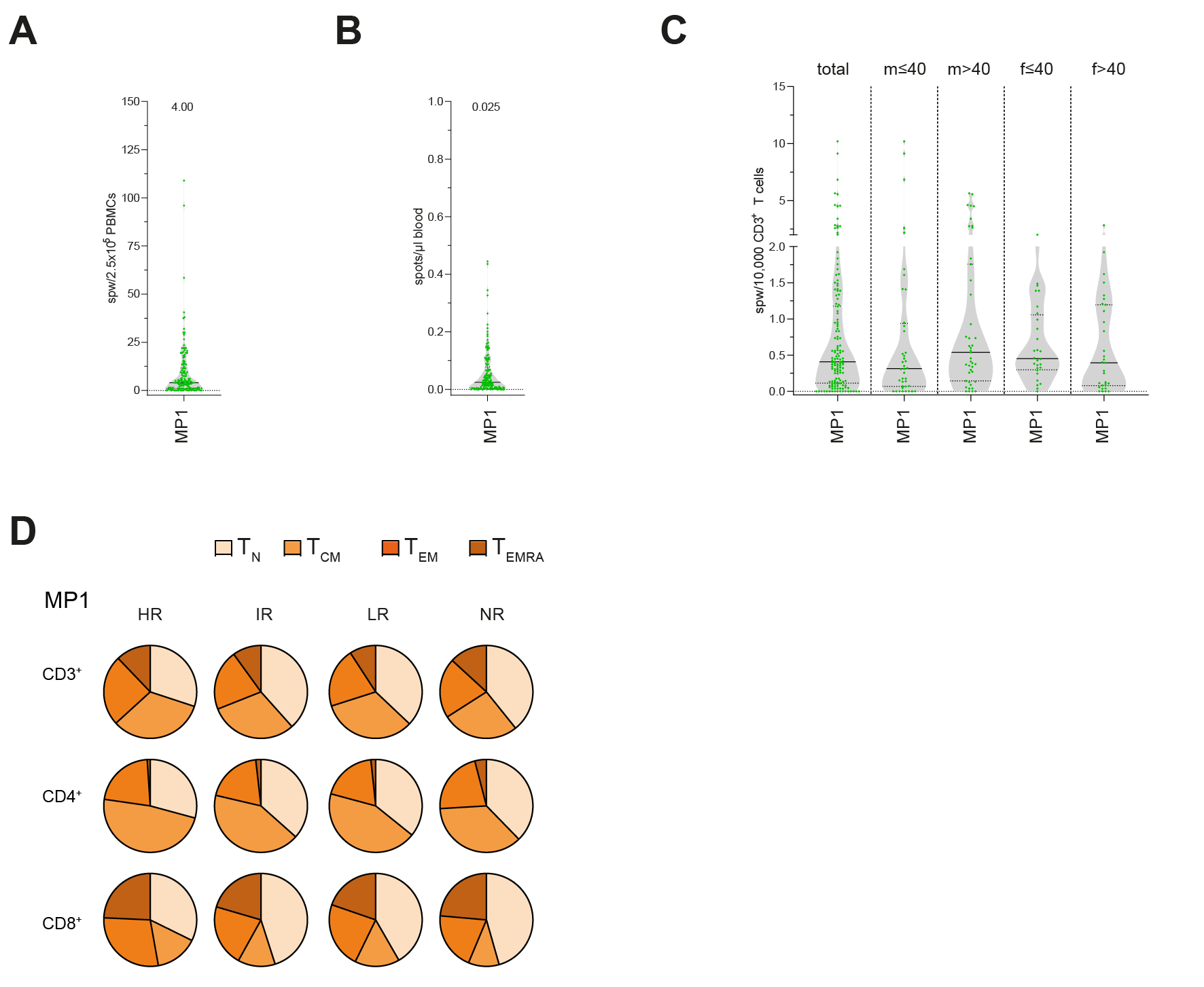


**Figure S11. Detection of Influenza A Virus (IAV)-specific memory T cells.** IAV_MP1-specific T cells in IAV-seropositive healthy donors (n=142) were detected by ELISpot and analyzed with respect to frequencies and memory subsets, as well as donor age and gender. (**A**) Frequencies of antigen-specific T cells among IAV-seropositive donors expressed as spw/2.5x10^5^ PBMCs. (**B**) Frequencies of antigen-specific T cells among IAV-seropositive donors expressed as spots/µl blood. (**A,B**) The number above each data set indicates the median. (**C**) Distribution of T-cell responses against IAV-specific peptide pool among IAV-seropositive donors in the whole group and donor groups divided by age and gender. (**D**) T-cell phenotypes for each IAV responder group. T_N_: naïve T cell (CD45RA^+^CD62L^+^), T_CM_: Central Memory T cells (CD45RA^-^CD62L^+^), T_EM_: Effector Memory T cells (CD45RA^-^CD62L^-^), T_EMRA_: Effector Memory T cell re-expressing CD45RA (CD45RA^+^CD62L^-^), HR: High Responder, IR: Intermediate Responder, LR: Low Responder, NR: Non-Responder.
